# Modified *ent*-Abietane Diterpenoids from the Leaves of *Suregada zanzibariensis*

**DOI:** 10.1021/acs.jnatprod.2c00147

**Published:** 2022-09-08

**Authors:** Thobias M. Kalenga, Jackson T. Mollel, Joanna Said, Andreas Orthaber, Jas S. Ward, Yoseph Atilaw, Daniel Umereweneza, Monica M. Ndoile, Joan J. E. Munissi, Kari Rissanen, Edward Trybala, Tomas Bergström, Stephen S. Nyandoro, Mate Erdelyi

**Affiliations:** † Chemistry Department, College of Natural and Applied Sciences, University of Dar es Salaam, P.O. Box 35061, Dar es Salaam, Tanzania; ‡ Department of Chemistry, College of Education, Mwalimu Julius K. Nyerere University of Agriculture and Technology, P.O. Box 976, Butiama, Tanzania; § Institute of Traditional Medicine, Muhimbili University of Health and Allied Sciences, P.O. Box 65001, Dar es Salaam, Tanzania; ∧ Department of Infectious Diseases/Virology, Institute of Biomedicine, Sahlgrenska Academy, University of Gothenburg, S-413 46 Gothenburg, Sweden; || Department of Chemistry − Ångström, Uppsala University, SE-751 20 Uppsala, Sweden; ∇ University of Jyvaskyla, Department of Chemistry, 40014 Jyväskylä, Finland; O Department of Chemistry − BMC, Uppsala University, SE-751 23 Uppsala, Sweden; ∞ Department of Chemistry, College of Science and Technology, University of Rwanda, P.O Box 3900, Kigali, Rwanda

## Abstract

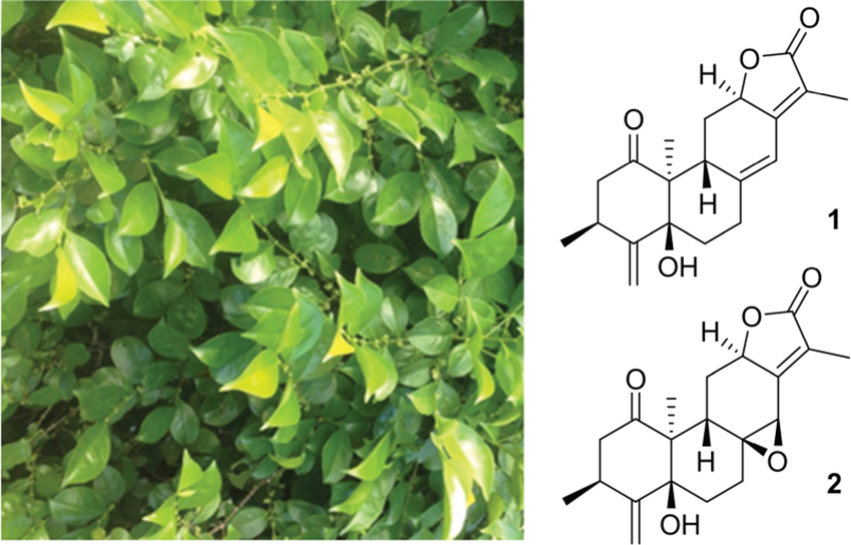

The leaf extract of *Suregada zanzibariensis* gave two new modified *ent*-abietane diterpenoids, zanzibariolides A (**1**) and B (**2**), and two known triterpenoids, simiarenol (**3**) and β-amyrin (**4**). The structures of the isolated compounds were elucidated based on NMR and MS data analysis. Single-crystal X-ray diffraction was used to establish the absolute configurations of compounds **1** and **2**. The crude leaf extract inhibited the infectivity of herpes simplex virus 2 (HSV-2, IC_50_ 11.5 μg/mL) and showed toxicity on African green monkey kidney (GMK AH1) cells at CC_50_ 52 μg/mL. The isolated compounds **1**–**3** showed no anti-HSV-2 activity and exhibited insignificant toxicity against GMK AH1 cells at ≥100 μM.

The genus *Suregada* (synonym *Gelonium*) belongs to the tribe Geloniae of the family Euphorbiaceae. It comprises 31 species that grow in the tropical and subtropical parts of Africa and Asia.^1−3^ Out of these, *Suregada zanzibariensis* Baill., *Suregada lithoxyla* (Pax & K. Hoffm.) Croizat, and *Suregada procera* (Prain) Croizat are found in Tanzania.^4^*S. zanzibariensis* is an evergreen shrub that may grow into small trees, up to 4–10 m tall. This species is also found in South Africa, Zimbabwe, Angola, Mozambique, Somalia, Kenya, and Madagascar.^1^ In Tanzania, the plant is known as “mndimu pori”, which means wild citrus plant in Swahili. In some parts of Tanzania, the root bark and stem bark of *S. zanzibariensis* are claimed to treat ancylostomiasis, whereas a tea made from its root bark is used to heal stomachache, gonorrhea, hernia, chest pain, pneumonia, and chicken pox and is also employed as a purgative. A decoction of its leaves is applied to treat skin infections, while its essential oil is reported to repel mosquitoes.^1^ A leaf extract of *S. zanzibariensis* was reported to exhibit cytotoxic activity against UACC62 melanoma, MCF-7 breast cancer, TK10 renal, and embryonic lung fibroblast (HELF) cells and to inhibit chloroquine-resistant (ENT36) and chloroquine-sensitive (K67) *Plasmodium falciparum* strains.^1,5^ So far, lactonized *ent*-abietane diterpenoids cytotoxic against the TK10, UACC62, and MCF-7 cancer cell lines have been reported from its stem bark extract.^1^ Herein, four further terpenoids have been isolated and identified and were then evaluated for activity against herpes simplex virus 2 (HSV-2) and for cytotoxicity against African green monkey kidney epithelial cells (GMK AH1).

## Results and Discussion

Repeated column chromatographic separation of the leaf extract (CH_3_OH–CH_2_Cl_2_, 7:3, v/v) of *S. zanzibariensis* yielded two new diterpenoids (**1** and **2**) along with the known triterpenoids simiarenol (**3**)^6^ and β-amyrin (**4**).^7^ The structures of the isolated compounds were determined by NMR spectroscopic and mass spectrometric analyses supported by single-crystal X-ray diffraction analysis.
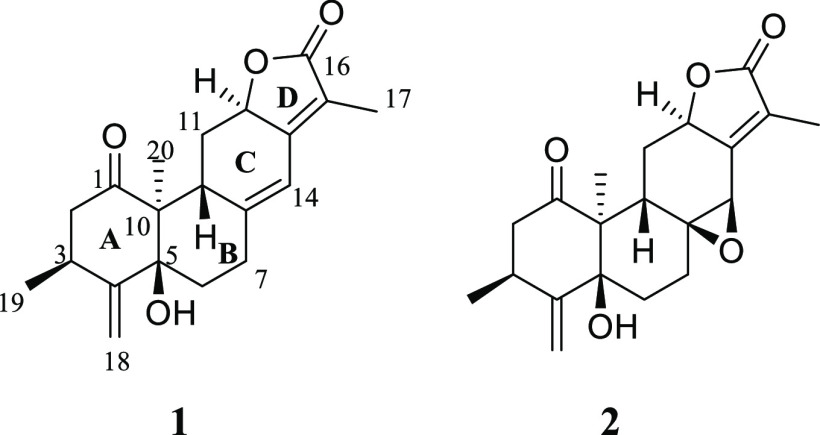


Compound **1**, [α]^24^_D_ +82.5 (*c* 0.03, CH_2_Cl_2_), was isolated as white crystals from a 1:1 mixture of CH_2_Cl_2_–isohexane. Its HRESIMS (Figure S8, Supporting Information) showed a molecular ion [M + H]^+^ at *m*/*z* 329.1754 (calcd 329.1753) consistent with the molecular formula, C_20_H_24_O_4_, suggesting nine double-bond equivalents. The compound gave a UV absorption at λ_max_ 264 nm, supporting the occurrence of an α,β-unsaturated carbonyl moiety, typical for diterpenoid lactones.^8,9^ Its strong IR absorption bands at 3460 and 1705 cm^–1^ indicated the presence of hydroxy and carbonyl groups, respectively. The ^1^H NMR spectrum (Table 1, Figure S1, Supporting Information) of **1** displayed a signal at δ_H_ 6.35 (H-14) corresponding to a trisubstituted alkene, signals at δ_H_ 5.16 (H-18a) and 5.09 (H-18b) typical of a terminal alkene, resonances at δ_H_ 4.72 (H-12) diagnostic for an oxymethine and at δ_H_ 3.52 (H-9) and 3.10 (H-3) indicative of two methines, signals typical for four pairs of diastereotopic methylene moieties [δ_H_ 2.96 (H-2a) and 2.19 (H-2b); 1.97 (H-6a) and 1.93 (H-6b); 2.70 (H-7a) and 2.32 (H-7b); 2.46 (H-11a) and 1.81 (H-11b)], and signals for three methyls [δ_H_ 1.83 (H-17), 1.32 (H-19), and 1.13 (H-20)]. The corresponding carbons were identified using the HSQC spectrum (Figure S4, Supporting Information). The ^13^C NMR spectrum (Table 1, Figure S2, Supporting Information) showed signals corresponding to 20 carbons, with chemical shifts compatible with a diterpenoid.^8,10,11^ Two carbonyl groups resonated at δ_C_ 210.9 (C-1) and 175.7 (C-16), which are typical for a ketone and a lactone, respectively.

**Table 1:** ^1^H and ^13^C NMR Spectroscopic Data (400 MHz, CDCl_3_, 25 °C) for Zanzibariolides A (**1**) and B (**2**)

	Zanzibariolide A (**1**)		Zanzibariolide B (**2**)	
position	δ_C_, type	δ_H_ (*J* in Hz)	δ_C_, type	δ_H_ (*J* in Hz)
1	210.9, C=O		210.4, C=O	
2	43.8, CH_2_	2.19, dd (14.0, 1.6)	43.2, CH_2_	2.26, dd (13.9, 1.6)
		2.96, dd (14.0, 9.0)		2.96, dd (13.9, 8.2)
3	38.9, CH	3.10, qdd (9.0,7.4, 1.6)	38.8, CH	3.08, qdd (8.2, 7.4, 1.6)
4	151.9, C		151.9, C	
5	80.4, C		79.9, C	
6	33.3, CH_2_	1.93, m	29.7, CH_2_	1.91, m
		1.97, m		2.09, ddd (14.3, 14.2, 4.5)
7	30.9, CH_2_	2.32, dd (13.3, 9.0)	28.1, CH_2_	1.40, m
		2.70, ddd (13.3, 5.4, 2.6)		2.49, ddd (14.1, 14.1, 5.4)
8	150.8, C		60.4, C–O	
9	36.7, CH	3.52, dd (8.8, 1.8)	33.2, CH	3.33, d (7.4)
10	58.0, C		55.7, C	
11	30.1, CH_2_	1.81, ddd (14.2, 8.8, 6.5)	26.22, CH_2_	1.63, ddd (13.9, 13.2, 7.4)
		2.46, dd (14.2, 6.5)		2.29, m
12	76.4, CH	4.72, ddd, (14.2, 6.5, 1.8)	76.0, CH	4.85, ddd (13.2, 5.8, 2.2)
13	156.0, C		155.2, C	
14	115.5, CH	6.35, br m	55.6, CH	3.73, s
15	117.1, C		128.9, C	
16	175.5, C=O		174.1, C=O	
17	8.6, CH_3_	1.83, d (1.7)	8.9, CH_3_	1.97, d (2.2)
18	114.2, CH_2_	5.09, br s	114.5, CH_2_	5.19, br m
		5.16, br s		5.22, br m
19	26.0. CH_3_	1.32, d (7.4)	25.6, CH_3_	1.33, d (7.4)
20	18.0, CH_3_	1.13, s	20.5, CH_3_	1.26, s

**Figure 1:**
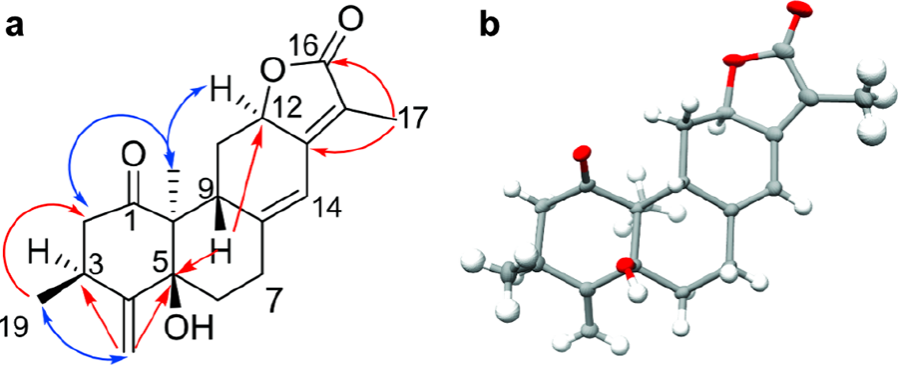
(a) Key HMBC (red) and NOESY (blue) correlations and (b) the single-crystal X-ray analysis-derived structure of zanzibariolide A (**1**) (thermal ellipsoids at the 50% probability level).

The HMBC (Figure 1a, Table S1, and Figure S5, Supporting Information) cross-peaks of H-20 (δ_H_ 1.13), H-2a/b (δ_H_ 2.96/2.19), H-3 (δ_H_ 3.10), and H-9 (δ_H_ 3.52) to C-1 (δ_C_ 210.9) as well as those of H-3 (δ_H_ 3.10) to C-4 (δ_C_ 151.9), C-5 (δ_C_ 80.4), and C-18 (δ_C_ 114.2) allowed the assignment of ring A. While the cross-peaks of H-19 (δ_H_ 1.32) to C-4 (δ_C_ 151.9) and H-18a/b (δ_H_ 5.16/5.09) to C-5 (δ_C_ 80.4) further supported the assignment of ring A, those of H-9 (δ_H_ 3.52) to C-5 (δ_C_ 80.4) and H-6a/b (δ_H_ 1.97/1.93) to C-4 (δ_C_ 151.9), C-8 (δ_C_ 150.8), and C-10 (δ_C_ 58.0) were used to deduce the linkage of ring A and B. Furthermore, the HMBC cross-peaks of the proton at δ_H_ 4.72 (H-12) to C-16 (δ_C_ 175.5) and C-14 (δ_C_ 115.5) and those of δ_H_ 1.83 (H-17) to C-16 (δ_C_ 175.5) and C-13 (δ_C_ 156.0) enabled the assignment of rings C and D. The COSY cross-peak of H-9 (δ_H_ 3.52) with H-11b (δ_H_ 1.81) and H-14 (δ_H_ 6.35) corroborated the proposed linkage of rings B and C. The H-14 signal (δ_H_ 6.35) appeared as a broad singlet and showed COSY cross-peaks to H-9 (δ_H_ 3.52), H-7a (δ_H_ 2.70), H-12 (δ_H_ 4.72), and H-17 (δ_H_ 1.83), linking rings B, C, and D. Further assignments were supported by the TOCSY spectrum of **1** (Figure S6, Supporting Information).

The relative configuration of **1** was deduced from coupling constants (Table 1) and NOESY correlations (Figure S7, Supporting Information). Thus, the NOEs observed between H-12 (δ_H_ 4.72) and H-20 (δ_H_ 1.13) suggested these protons to be *syn* oriented. A weak positive Cotton effect was observed for the π → π* at 320 nm, a negative Cotton effect was observed for the n → π* transition at 287 nm, and a strong negative Cotton effect at ca. 214 nm for the ^1^La electronic transition was seen in the electronic circular dichroism (ECD) spectrum of **1** (Figure S17, Supporting Information). Single-crystal X-ray diffraction analysis using Cu Kα radiation was performed (Figure S18, Supporting Information), establishing unambiguously the absolute configuration of **1** as 3*S*,5*S*,9*S*,10*S*,12*R* (Figure 1b). Based on the spectroscopic data obtained, this new compound, zanzibariolide A (**1**), was characterized as the *ent*-abietane (3*S*,5*S*,9*S*,10*S*,12*R*)-5-hydroxy-3,10,15-trimethyl-4-methylene-2,3,6,7, 9,11,12,14-decahydrophenanthro[3,2-*b*]furan-1,16-dione.

Compound **2**, [α]^24^_D_ −87.5 (*c* 0.03, CH_2_Cl_2_), was isolated as white crystals and assigned the molecular formula C_20_H_24_O_5_ based on HRESIMS ([M + H]^+^ at *m*/*z* 345.1702, calcd 345.1702, Figure S16, Supporting Information) and NMR (Table 1) analyses. This molecular formula indicated nine double-bond equivalents. Its UV absorption at λ_max_ 270 nm suggested the presence of an α,β-unsaturated carbonyl moiety. Strong IR absorption bands were observed at 3456 and 1710 cm^–1^ that were in line with the presence of hydroxy and carbonyl groups, respectively. The NMR spectroscopic data of **2** (Table 1, Figures S9–S15, Supporting Information) resembled those of compound **1**, except for the differences associated with an epoxy moiety, which was established to be at C-8 and C-14 of ring C. This epoxy group was identified by the presence of signals at δ_C_ 60.4 (C-8) and δ_C_ 55.6 (C-14), replacing those at δ_C_ 150.8 and δ_C_ 115.5 observed for compound **1**. Therefore, the ^1^H NMR spectrum of **2** contained a signal at δ_H_ 3.73 (H-14), compatible with an oxymethine functionality, instead of the olefinic proton signal at δ_H_ 6.35 that was observed for **1**.

**Figure 2:**
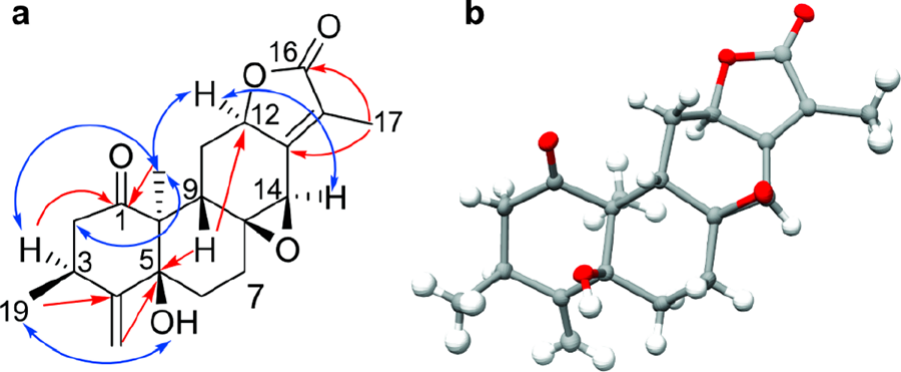
(a) Key HMBC (red) and NOESY (blue) correlations and (b) the solid-state structure for zanzibariolide B (**2**) (thermal ellipsoids set at the 50% probability level).

Similar to **1**, the ^1^H NMR spectrum of **2** displayed signals typical for geminal protons of a terminal alkene at δ_H_ 5.22/5.19 (H-18a/18b) and for an oxymethine proton at δ_H_ 4.85 (H-12), two methine protons at δ_H_ 3.33 (H-9) and δ_H_ 3.08 (H-3), four pairs of diastereotopic protons at δ_H_ 2.96/2.26 (H-2a/2b), 2.09/1.91 (H-6a/6b), 2.49/1.40 (H-7a/7b), and 2.29/1.63 (H-11a/11b), and three methyl protons at δ_H_ 1.97 (H-17), 1.33 (H-19), and 1.26 (H-20). Its ^13^C NMR spectrum (Table 1, Figure S10, Supporting Information) consisted of signals corresponding to 20 carbons, which is in agreement with a diterpenoid skeleton.^8,10,11^ Similar to the HMBC spectrum of compound **1**, that of **2** (Figure 2, Figure S13 and Table S1, Supporting Information) showed cross-peaks from H-20 (δ_H_ 1.26), H-2a/b (δ_H_ 2.96/2.26), H-3 (δ_H_ 3.08), and H-9 (δ_H_ 3.33) to C-1 (δ_C_ 210.4), which together with the HMBC cross-peaks from H-3 to C-4 (δ_C_ 151.9), C-5 (δ_C_ 79.9), and C-18 (δ_C_ 114.5) aided in the assignment of ring A. In addition, the cross-peaks of H-12 (δ_H_ 4.85) to C-16 (δ_C_ 174.1) and of C-14 (δ_C_ 55.4) and H-17 (δ_H_ 1.97) to C-16 (δ_C_ 174.1) and C-13 (δ_C_ 155.2) confirmed the assignment of rings C and D. The HMBC cross-peak of H-9 to C-5 (δ_C_ 79.9), C-14 (δ_C_ 55.4), and C-12 (δ_C_ 76.0) along with the long-range ^5^*J*_H12–H17_ coupling observed in the COSY spectrum (Figure S11, Supporting Information) supported the proposed linkage of rings B and C.

The relative configuration of **2** was determined based on NOE observations (Figure 2a, Figure S15, Supporting Information) and scalar couplings (Table 1). Thus, the strong NOE cross-peak between H-12 (δ_H_ 4.85) and H-20 (δ_H_ 1.26) suggested these protons to be *syn*-oriented, similar to **1**. The ECD spectrum of **2** (Figure S17, Supporting Information) showed a positive Cotton effect for the π → π* transition at ca. 293 nm, a positive Cotton effect for the n → π* transition at ca. 256 nm, a negative Cotton effect at ca. 241 nm for the n → π*, and a weak positive Cotton effect at 210 nm for the ^1^La electronic transition. This is different from that observed for **1** and for other previously reported *ent*-abietane diterpenoids.^1,12^ Single-crystal X-ray diffraction analysis using Cu Kα radiation (Figure 2b) established unambiguously the absolute configuration of **2** as 3*S*,5*S*,8*S*,9*S*,10*S*,12*R,*14*R*. Based on the above spectroscopic analyses, this new compound, zanzibariolide B (**2**), was characterized as the *ent*-abietane (3*S*,5*S*,8*S*,9*S*,10*S*,12*R,*14*R*)-5-hydroxy-8,14-epoxy-3,10,15-trimethyl-4-methylene-2,3,6,7,9,11,12,14-decahydrophenanthro[3,2-*b*]furan-1,16-dione.

**Scheme 1:**
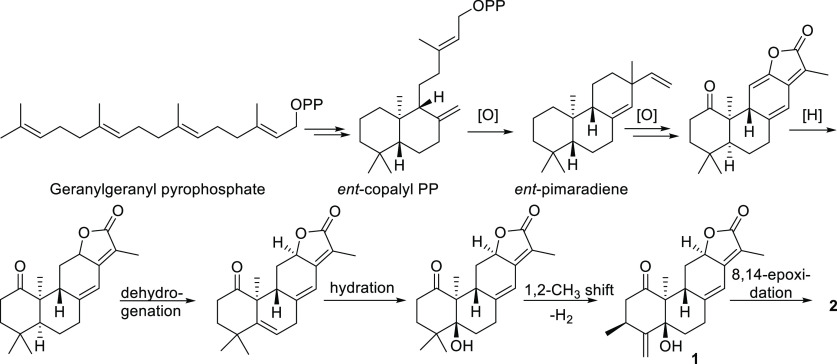
Plausible Biogenesis of Zanzibariolides A (**1**) and B (**2**)

The proposed biogenesis of **1** and **2** is shown in Scheme 1. The terminal double bond at C-4 is proposed to arise through an enzymatic 1,2-methyl shift, either of CH_3_-18 or CH_3_-19, from C-4 to C-3, followed by dehydrogenation. Such a methyl shift is a common phenomenon in terpene biosynthesis.^13^

**Figure 3:**
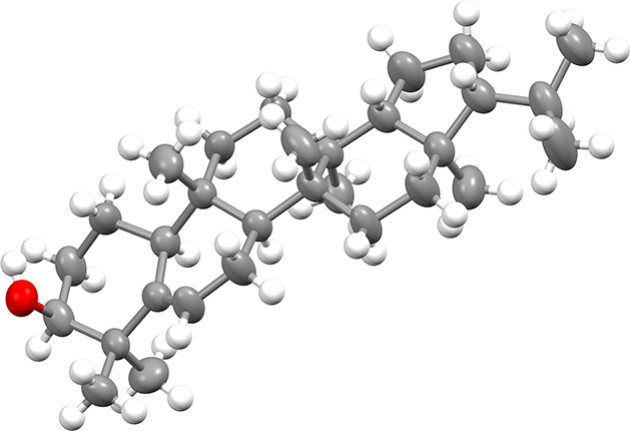
Solid-state structure of simiarenol (**3**) (thermal ellipsoids at the 50% probability level).

*ent*-Abietane diterpenoids have been reported from various plants,^11,14,15^ including also *Suregada* species.^1,4,8,11,16,24^ However, modified *ent*-abietane diterpenoids with a terminal olefinic bond at C-4, as in compounds **1** and **2**, are rare.^17,18^ The structures of the isolated known triterpenoids, simiarenol (**3**),^19,20^ and β-amyrin (**4**)^7,21^ were confirmed by comparison of their spectroscopic data (Figures S18–S30, Supporting Information) to those reported in the literature.^7,19,21^ The relative configuration of **3** was confirmed by single-crystal X-ray diffraction analysis (Figure 3).

**Figure 4:**
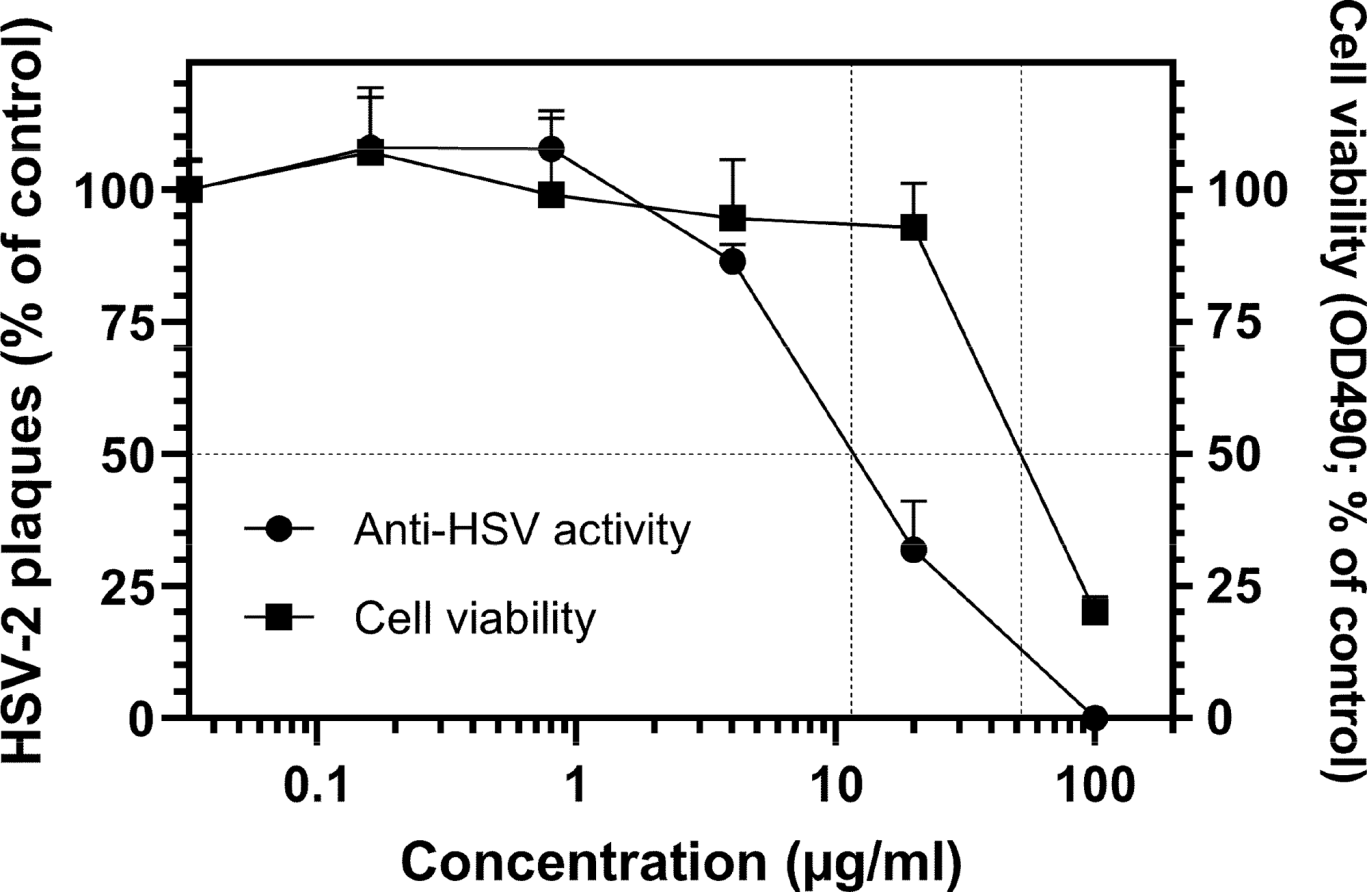
Anti-HSV-2 activity and cytotoxicity of the leaf crude extract of *S. zanzibariensis.* To test for anti-HSV-2 activity, the extract at indicated concentrations and 100 plaque-forming units of HSV-2 were added to GMK AH1 cells, and after incubation for 3 days, the cells were stained with crystal violet to visualize the viral plaques. The results are expressed as % of the number of viral plaques detected with the extract relative to those found in nontreated DMSO controls. For the cytotoxicity assay, GMK AH1 cells were incubated with indicated concentrations of the extract for 3 days, prior to the addition of the CellTiter 96 AQueous reagent (Promega, Madison, WI, USA) and recording the absorbance at 490 nm. The results are expressed as % of absorbance recorded with the extract relative to that found in nontreated DMSO controls. The data shown are means of four replicates from the two separate experiments.

**Figure 5:**
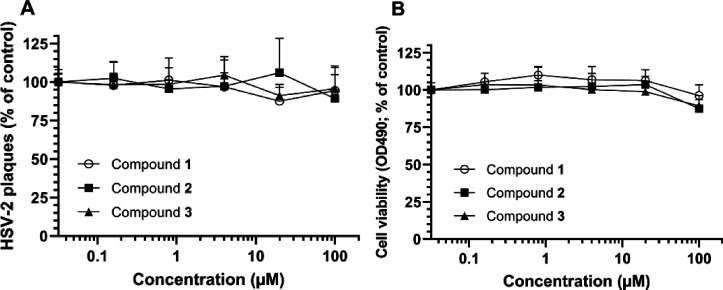
Anti-HSV-2 activity (A) and toxicity for GMK AH1 cells (B) of Zanzibariolide A (**1**) and B (**2**), and of Simiarenol (**3**). For details, see the legend to Figure 4. Each data point is a mean of four replicates from two separate experiments.

The anti-HSV-2 activity and the cytotoxicity of the leaf crude extract and of compounds **1**–**3** are shown in Figures 4 and 5, respectively. The crude extract exhibited anti-HSV-2 activity with an IC_50_ of 11.5 μg/mL, while it reduced GMK AH1 cell viability by 50% (CC_50_) at 52 μg/mL (Figure 4), giving a selectivity index CC_50_/IC_50_ = 4.5. Hence, some components of the crude extract may possess anti-HSV-2 activity at noncytotoxic concentrations. Encouraged by these data, compounds **1**–**3**, purified from this extract, were tested for their bioactivities. None exhibited anti-HSV-2 activity at a concentration up to 100 μM (Figure 5a). Compounds **1**–**3** were evaluated also for their ability to inhibit infection of A549 cells by the tick-borne encephalitis virus (TBEV) and infection of HeLa cells by the human rhinovirus type 2 (HRV-2) (page S23, Supporting Information). Under the concentration range tested (0.032–100 μM) compounds **1**–**3** exhibited no anti-TBEV nor HRV-2 activities. These compounds showed very little or no toxicity for GMK AH1 cells at ≥100 μM (Figure 5b). Nonetheless, the potential cytotoxic effect of the leaf crude extract at 100 μg/mL raises safety concerns as the concoction of leaves from the plant is used in folk medicine for various ailments.^1,3^ On the other hand, compound **3** has previously been reported to exhibit significant activity against α-glucosidase^22^ and to be toxic (IC_50_ 1.78 μM) against human acute monocytic leukemia cells (THP-1).^23^ Compound **4** was not tested for anti-HSV-2 activity, as it was isolated in low amount; however, it is known to exhibit significant anti-inflammatory activity by inhibition of PGE2 and IL-6 secretion.^21^

## Experimental Section

### General Experimental Procedures

Optical rotations were determined using a 341 LC OROT polarimeter at 589 nm and 24.0 °C, whereas ECD spectra were acquired on a JASCO J-810, Rev.1.00, spectropolarimeter. UV spectra were obtained using CH_3_OH as the solvent on a Shimadzu UV-1650PC UV/vis spectrophotometer. Infrared (IR) spectra were recorded on a PerkinElmer Spectrum FT-IR spectrometer using liquid samples. NMR spectra were acquired either on an Agilent 400MR 400 MHz spectrometer equipped with a OneNMRProbe or on a Bruker Avance Neo 600 MHz spectrometer equipped with a TCI cryogenic probe and were processed using MestreNova (v14.0.0). Chemical shifts were referenced to the residual of carbon and proton signals of the deuterated solvents (CDCl_3_ δ_H_ 7.26 and δ_C_ 77.16) as internal standard. Assignments were based on 1D (^1^H and ^13^C) and 2D (COSY, HSQC, HMBC, TOCSY, and NOESY) NMR spectra. Mass spectra were acquired on a Waters Micromass ZQ Multimode Ionization ESCI in ESI mode, connected to an Agilent 1100 series gradient pump system and a C18 Atlantis T3 column (3.0 × 50 mm, 5 μm), and using Milli-Q H_2_O–MeOH (5:95 to 95:5, with 1% HCO_2_H and a flow rate of 0.75 mL/min over 6 min). HRESIMS spectra were obtained with a Q-TOF-LC/MS spectrometer using a 2.1 × 30 mm 1.7 μM RPC_18_ and H_2_O–CH_3_CN gradient (5:95–95:5 in 0.2% formic acid, v/v) at Sternhagen Analys Lab AB, Gothenburg, Sweden. Thin layer chromatography (TLC) was performed on silica gel 60 F_254_ (Merck, Darmstadt, Germany) using precoated aluminum plates to monitor isolation processes. TLC plates were visualized under UV light (254 and 366 nm) and by spraying with an anisaldehyde reagent (prepared by mixing 3.5 mL of 4-anisaldehyde with 2.5 mL of concentrated sulfuric acid, 4 mL of glacial acetic acid, and 90 mL of methanol) followed by heating (80–100 °C). Column chromatography was run on silica gel 60 (230–400 mesh), whereas gel filtration on Sephadex LH-20 (GE Healthcare).

### Plant Material

The leaves of *Suregada zanzibariensis* were collected in May 2017 from Umasaini bushland near Mng’ongo Bridge (6°25′20.814″ S; 38°42′13.722′′ E at an elevation of 40 m altitude) in Fukayosi village, Bagamoyo District, Pwani Region in Tanzania. The plant was identified by Mr. F. M. Mbago, a senior taxonomist of the Herbarium, Botany Department, University of Dar es Salaam, and the specimens were deposited with voucher number FMM 3811 at the Herbarium, Botany Department, University of Dar es Salaam.

### Extraction and Isolation

The air-dried leaves of *S. zanzibariensis* were ground to a fine powder to obtain a 1603 g sample, which was soaked three times in 3 L of CH_3_OH–CH_2_Cl_2_ (7:3) at room temperature for 48 h, yielding a total of 74 g of crude extract after evaporation under reduced pressure at 40 °C. The crude extract (71 g) was adsorbed onto silica gel (1:1) and loaded on a silica gel 60 (230–400 mesh) column. Gravitational elution was performed with a gradient of increasing polarity using EtOAc (0–100%) in isohexane, by collecting 92 fractions. Chromatographic separation at 30% EtOAc–isohexane gave fractions 26–34, which were combined and purified on a Sephadex LH-20 column (CH_3_OH–CH_2_Cl_2_, 1:1) to obtain a subfraction that was further separated with preparative TLC (silica gel) with EtOAc–isohexane (1:5) to afford β-amyrin^7^ (**4**, 4 mg). Combined fractions 38–46 that were obtained at 40% from column chromatography were purified on a Sephadex LH-20 column (CH_3_OH–CH_2_Cl_2_, 2:3) and further washed with isohexane to give simiarenol^6^ (**3**, 16 mg); 6 mg of this sample was crystallized from CH_2_Cl_2_–isohexane (1:1). Fractions 48–55 (obtained with 50% EtOAc–isohexane) were combined and washed with isohexane and further crystallized from CH_2_Cl_2_–isohexane (1:1) to afford zanzibariolide A (**1**, 236 mg) as white needle-like crystals. Furthermore, the combined fractions 62–84 eluted with 60–80% were washed with isohexane and crystallized from CH_2_Cl_2_–isohexane (1:1), affording zanzibariolide B (**2**, 1800 mg) as white needle-like crystals.

#### Zanzibariolide A (**1**):

White crystals; [α]^24^_D_ +82.5 (*c* 0.03, CH_2_Cl_2_); UV (CH_2_Cl_2_) λ_max_ 264 nm; ECD (*c* 0.025, CH_3_OH) λ_max_ (Δε) 310 (11), 287 (−34.1), 214 (−186.0); IR ν_max_ 3460, 1740 cm^–1^; ^1^H and ^13^C NMR, see Table 1; HRESIMS *m*/*z* 329.1754 [M + H]^+^ (calcd 329.1753 for [C_20_H_24_O_4_ + H]^+^).

#### Zanzibariolide B (**2**):

White crystals; [α]^24^_D_ −87.5 (*c* 0.03 CH_2_Cl_2_); UV (CH_2_Cl_2_) λ_max_ 270 nm; ECD (*c* 0.05, CH_3_OH) λ_max_ (Δε) 293 (18.7), 256 (31.0), 241 (−6.3); IR ν_max_ 3456, 1752, cm^–1^; ^1^H and ^13^C NMR, see Table 1; HRESIMS *m*/*z* 345.1702 [M + H]^+^ (calcd 345.1702 for [C_20_H_24_O_5_ + H]^+^).

### X-ray Crystal Structure Analysis

Single-crystal X-ray data for **1** and **2** were measured using a Rigaku SuperNova dual-source Oxford diffractometer equipped with an Atlas detector using mirror-monochromated Cu Kα (λ = 1.541 84 Å) radiation. The data collection and reduction were performed using the program CrysAlisPro,^24^ and a numerical absorption correction based on Gaussian integration was applied The structure was solved with intrinsic phasing (ShelXT)^25^ and refined by full-matrix least-squares on *F*^2^ using the Olex2 software,^26^ which utilizes the ShelXL module.^27^ Anisotropic displacement parameters were assigned to non-H atoms. All C–H hydrogen atoms were refined using riding models with a *U*_eq_(H) of 1.5*U*_eq_(C) for methyl groups and a *U*_eq_(H) of 1.2*U*_eq_(C) for all other C–H groups. Single-crystal X-ray diffraction measurements for compound **3** were performed using graphite-monochromatized Mo Kα radiation (λ = 0.710 73) using a Bruker D8 APEX-II equipped with a CCD camera. Data reduction was performed with SAINT. Absorption corrections for the area detector were performed using SADABS. The structure was solved by direct methods and refined by full-matrix least-squares techniques against *F*^2^ using all data (ShelXT, ShelXS).^27^ All non-hydrogen atoms were refined with anisotropic displacement parameters. Hydrogen atoms were constrained in geometric positions to their parent atoms using OLEX2.^28^ Diffuse contribution to diffraction in **3** was accounted for by using solvent masking.^29^ The X-ray structures of **1** (CCDC 2181946), **2** (CCDC 2181947), and **3** (CCDC 2118304) have been deposited at the Cambridge Crystallographic Data Centre. Copies of the data can be obtained, free of charge, on application to Director, CCDC, 12 Union Road, Cambridge CB2 IEZ, UK (fax: + 44-(0)1223-336033 or email: deposit@ccdc.cam.ac.uk).

#### Crystal data for **1**:

C_20_H_24_O_4_, M = 328.39, colorless block, orthorhombic, space group *P*2_1_2_1_2_1_, *a* = 8.4509(1) Å, *b* = 10.7590(1) Å, *c* = 18.4968(2) Å, *V* = 1681.79(3) Å^3^, *Z* = 4, *D*_calc_ = 1.297 g cm^–3^, *F*(000) = 704, μ = 0.72 mm^–1^, *T* = 120.0(1) K, θ_max_ = 76.4°, 3400 total reflections, 3328 with *I*_o_ > 2σ(*I*_o_), *R*_int_ = 0.020, 3400 data, 223 parameters, no restraints, GooF = 1.03, *R*_1_[*I*_o_ > 2σ(*I*_o_)] = 0.028 and *wR*_2_ = 0.075, 0.22 < dΔρ < −0.14 e Å^–3^, Flack = 0.07(6), CCDC 2181946.

#### Crystal data for **2**:

C_20_H_24_O_5_, M = 344.39, colorless plate, monoclinic, space group *P*2_1_, *a* = 6.2085(1) Å, *b* = 16.2459(3) Å, *c* = 8.2684(1) Å, β = 93.354(2)°, *V* = 832.54(2) Å^3^, *Z* = 2, *D*_calc_ = 1.374 gcm^–3^, *F*(000) = 368, μ = 0.80 mm^–1^, *T* = 120.0(1) K, θ_max_ = 76.4°, 3374 total reflections, 3278 with *I*_o_ > 2σ(*I*_o_), *R*_int_ = 0.022, 3374 data, 232 parameters, 1 restraint, GooF = 1.05, *R*_1_[*I*_o_ > 2σ(*I*_o_)] = 0.029 and *wR*_2_ = 0.075, 0.19 < dΔρ < −0.14 e Å^–3^, Flack = −0.04(7), CCDC 2181947.

#### Crystal data for **3**:

C_30_H_50_O (M = 426.70 g/mol), trigonal, space group *R*3, *a* = 35.206(6) Å, *c* = 7.3631(14) Å, *V* = 7903(3) Å^3^, *Z* = 9, *T* = 180.15 K, μ(Mo Kα) = 0.047 mm^–1^, *D*_calc_ = 0.807 g/cm^3^, 30 808 reflections measured (4.008° ≤ 2θ ≤ 50.236°), 6228 unique (*R*_int_ = 0.0808, *R*_sigma_ = 0.0839) which were used in all calculations. The final *R*_1_ was 0.0592 (*I* > 2σ(*I*)) and *wR*_2_ was 0.1334 (all data), CCDC 2118304.

### Antiviral Assay

African green monkey kidney epithelial cells^30^ were employed for screening of antiviral and cytotoxic activities of both crude extracts and pure compounds isolated therefrom. The HSV-2 333 strain^31^ was used. An HSV-2 plaque reduction assay was used to determine the effects of the plant extract and compounds on HSV-2 infectivity in GMK AH1 cells.^32^ Briefly, the plant extract and all tested compounds were solubilized in DMSO, and the stocks (10 mg/mL) were stored at −20 °C. Prior to the assay, the test samples were subjected to serial 5-fold dilutions in Eagle’s minimum essential medium supplemented with 1% penicillin/streptomycin and 1% l-glutamine stocks (EMEM-M) to obtain a concentration range 1.6–1000 μg/mL (extract) or 1.6–1000 μM (compounds). The control sample comprised various concentrations of DMSO solvent. The GMK AH1 cells were seeded in 24-well plates, and confluent, 3-day-old monolayers (ca. 3.7 × 10^5^ cells/well) were used. The supernatant culture medium was removed, the cells were rinsed once with 200 μL of EMEM-M medium, and 400 μL of fresh EMEM-M was added. Then, the cells in duplicate wells received 50 μL of serial 5-fold dilutions of extract or compounds and after gentle shaking were left at 37 °C in a humidified atmosphere comprising 5% CO_2_ (the CO_2_ incubator). Subsequently, 50 μL of EMEM-M medium comprising 100 plaque forming units of HSV-2 333 strain was added to each well, and following gentle shaking, the cells were left in the CO_2_ incubator for 90 min. Then, the supernatant medium was removed, and the cells were overlaid with 750 μL of a 1% solution of methyl cellulose in EMEM-M (supplemented with 2% fetal calf serum) that contained the same concentrations of the test extract or compounds. Following incubation of cells for 3 days in the CO_2_ incubator, the overlay medium was removed and the cells were stained with 1% crystal violet solution to visualize the viral plaques.

Cytotoxicity of the test extract or compounds for GMK AH1 cells was assayed as described by Said et al.^32^ Briefly, 3-day-old monolayer cultures of GMK AH1 cells growing in 96-well cluster plates were used. The culture medium was removed, the cells were rinsed with 200 μL of EMEM-M medium, and 50 μL of fresh EMEM was added. Subsequently, 50 μL of EMEM-M comprising the test samples at 5-fold dilutions was added in duplicate wells. The final concentrations of the extract and compounds were 100, 20, 4, 0.8, 0.16, and 0 (DMSO control) μg/mL (extract) or μM (compounds). Following incubation of cells with the test samples for 3 days in the CO_2_ incubator, 15 μL of the CellTiter 96 AQueous One Solution reagent (Promega, Madison, WI, USA) was added. After shaking, the cells were left in the CO_2_ incubator for a further 1 h, and absorbance at 490 nm was recorded.

## Supporting Information Available

NMR and MS data for the isolated compounds (PDF)
X-ray crystallographic data for compound **1** (CIF)
X-ray crystallographic data for compound **2** (CIF)
X-ray crystallographic data for compound **3** (CIF)

## Supplementary Material

np2c00147_si_001.pdf

np2c00147_si_002.cif

np2c00147_si_003.cif

np2c00147_si_004.cif

## Notes

The original MS and NMR spectra for all compounds, along with the corresponding NMReDATA^33,34^ for the new compounds **1** and **2**, are freely available on Zenodo as DOI 10.5281/zenodo.5920668.
