# Supplementary material for: Modified ent-Abietane Diterpenoids from the Leaves of Suregada zanzibariensis
Source: J Nat Prod. 2022 Sep 8;85(9):2135–41. doi: 10.1021/acs.jnatprod.2c00147 (PMC9513791; doi:10.1021/acs.jnatprod.2c00147)
Supplement: Supplementary file 1 — np2c00147_si_001.pdf [file np2c00147_si_001.pdf]

SUPPORTING INFORMATION

Modified *ent*-abietane Diterpenoids from the Leaves of *Suregada zanzibariensis*

Thobias M. Kalenga, Jackson T. Mollel, Joanna Said, Andreas Orthaber, Jas. S. Ward, Yoseph Atilaw, Daniel Umereweneza, Monica M. Ndoile, Joan J. E. Munissi, Kari Rissanen, Edward Trybala, Tomas Bergström, Stephen S. Nyandoro, and Mate Erdelyi

Table of Contents

1. Natural products isolated from the leaves of *Suregada zanzibariensis* S2
2. Spectroscopic data of zanzibariolide A (**1**) S2
3. Spectroscopic data of zanzibariolide B (**2**) S6
4. Spectroscopic data of Simiarenol (**3**) S12
5. Spectroscopic data of β-amyrin (**4**) S16
6. X-ray crystallography of zanzibariolide A (**1**), zanzibariolide B (**2**) and simiarenol (**3**) S21
7. Anti-tick-borne encephalitis virus (TBEV) and anti-human rhinovirus 2 activity assays S23

The original MS and NMR spectra for all compounds, along with the corresponding NMReDATA for the new compounds **1-2** are freely available on Zenodo as DOI:10.5281/zenodo.5920668.

1. Natural products isolated from the leaves of *Suregada zanzibariensis*

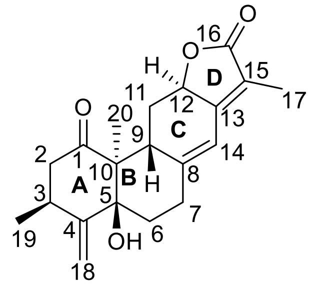

1

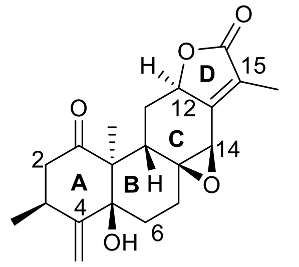

2

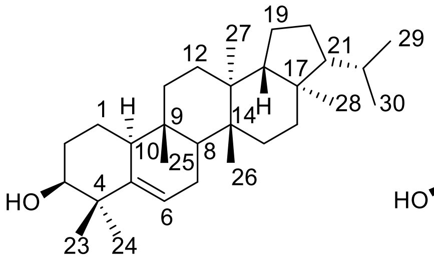

3

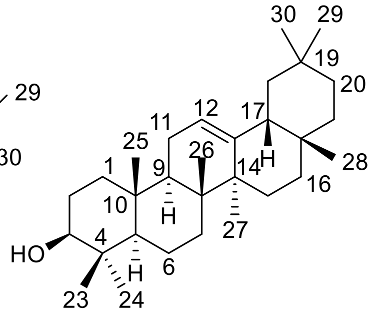

4

2. Spectroscopic data of zanzibariolide A (1)

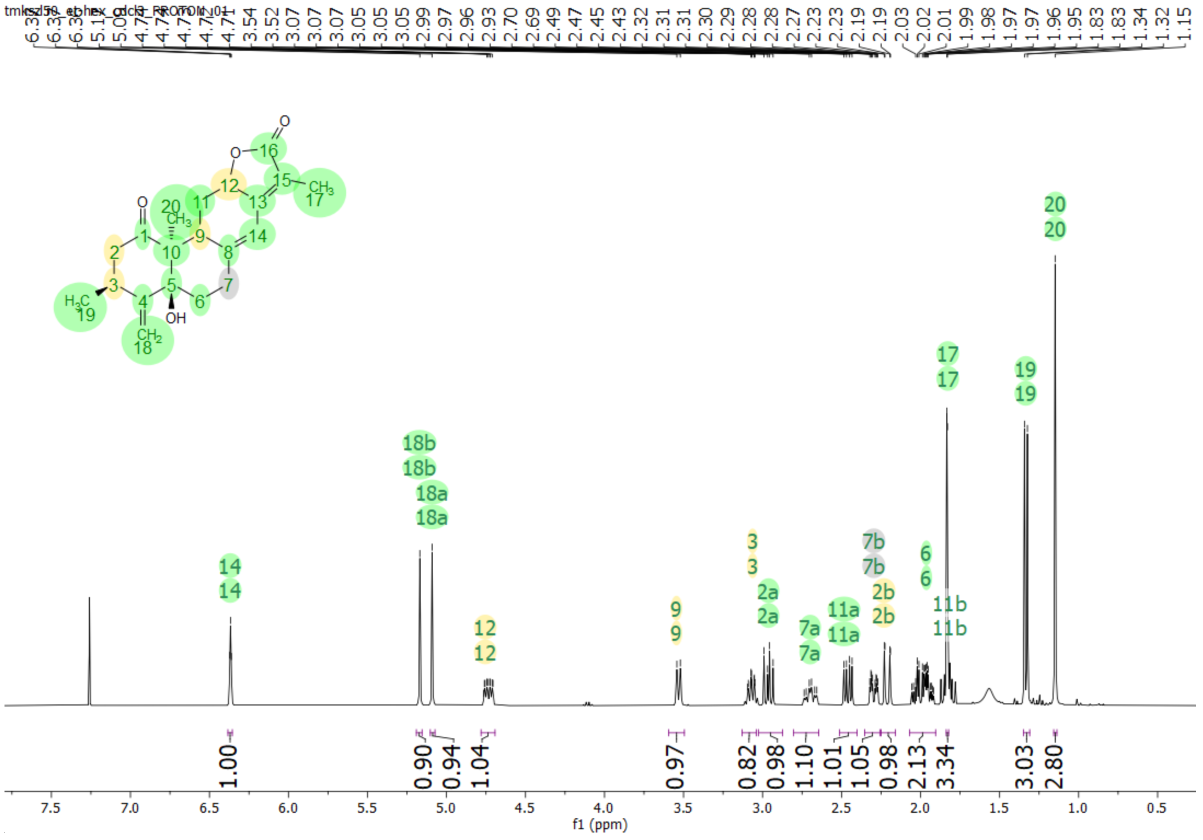

Figure S1: ^{1}H NMR Spectrum (400 MHz, $CDCl_3$, 25 ºC) of Zanzibariolide A (**1**)

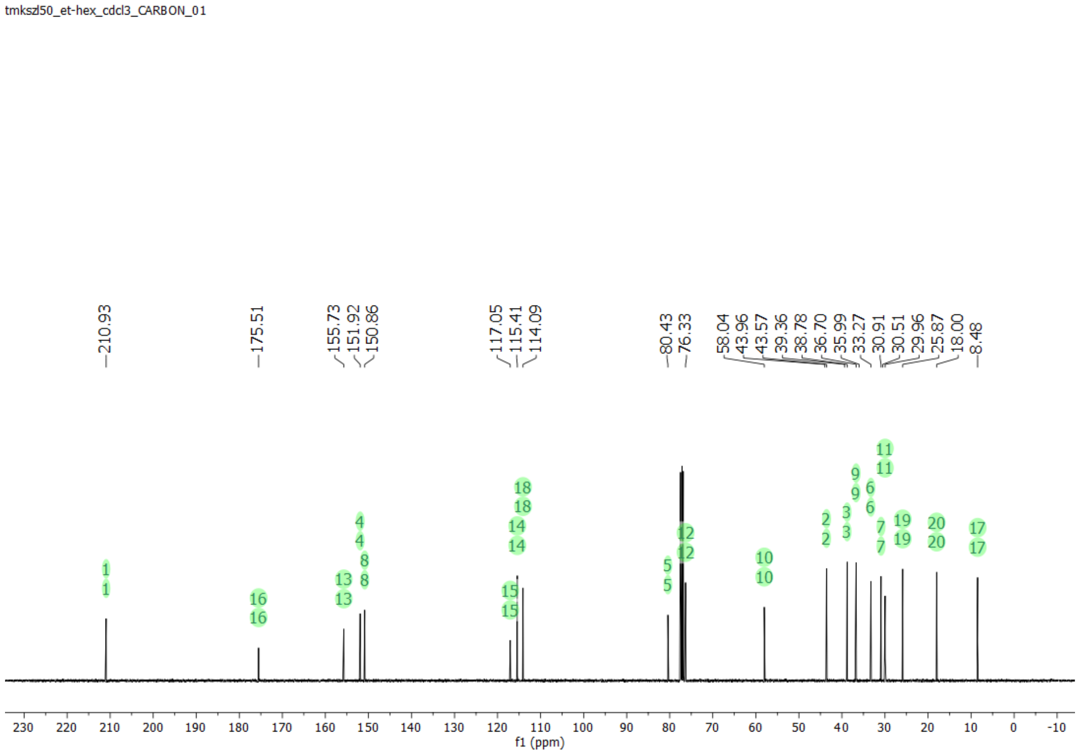

Figure S2: ^{13}C NMR Spectrum (100 MHz, $CDCl_3$, 25 ºC) of Zanzibariolide A (**1**)

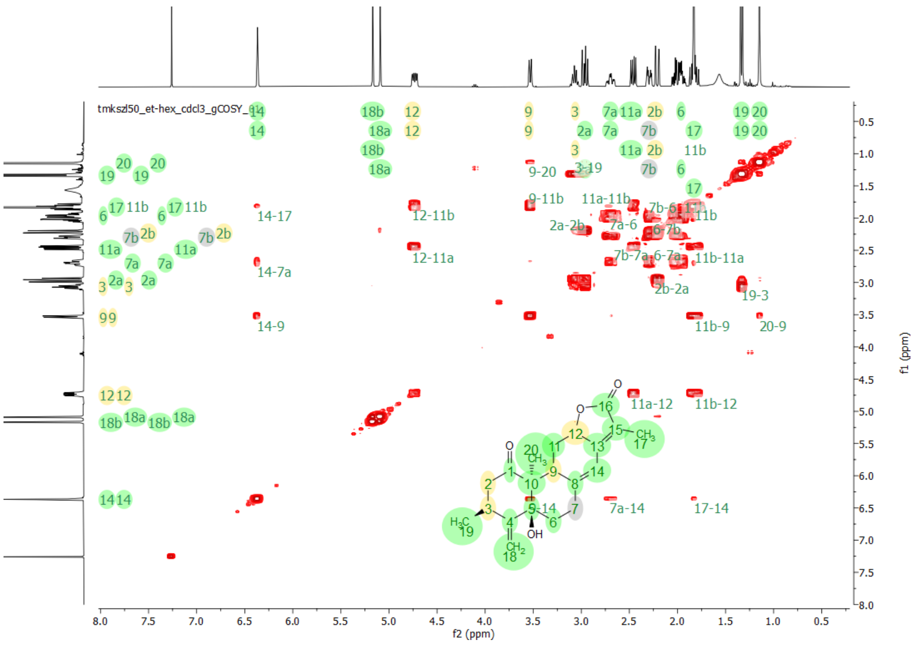

Figure S3: COSY Spectrum (400 MHz, $CDCl_3$, 25 ºC) of Zanzibariolide A (**1**)

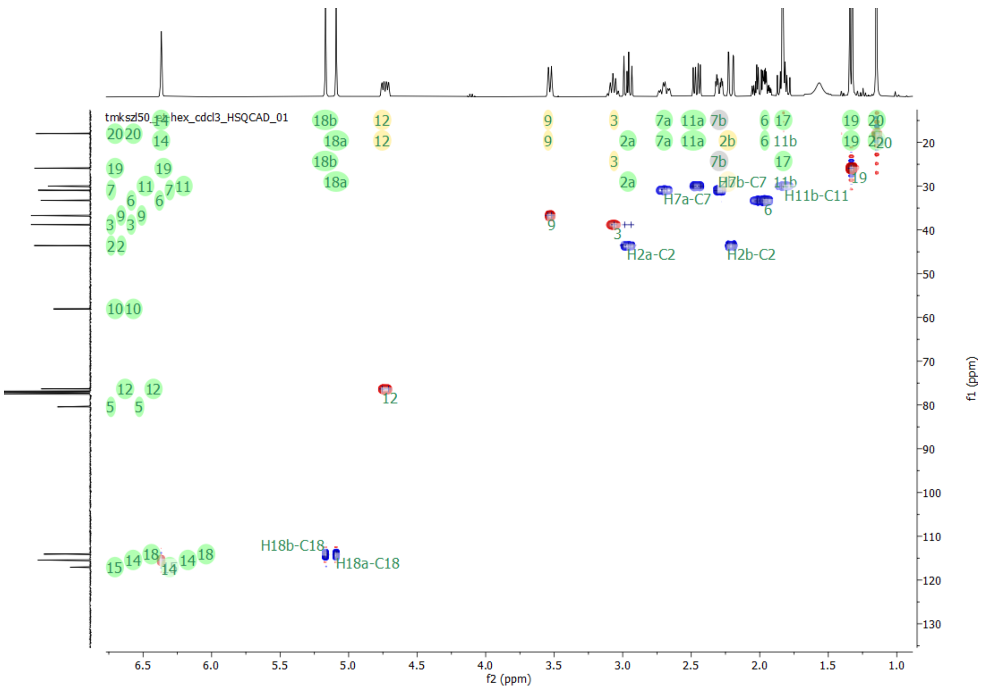

Figure S4: HSQC Spectrum (400/100 MHz, $CDCl_3$, 25 ºC) of Zanzibariolide A (**1**)

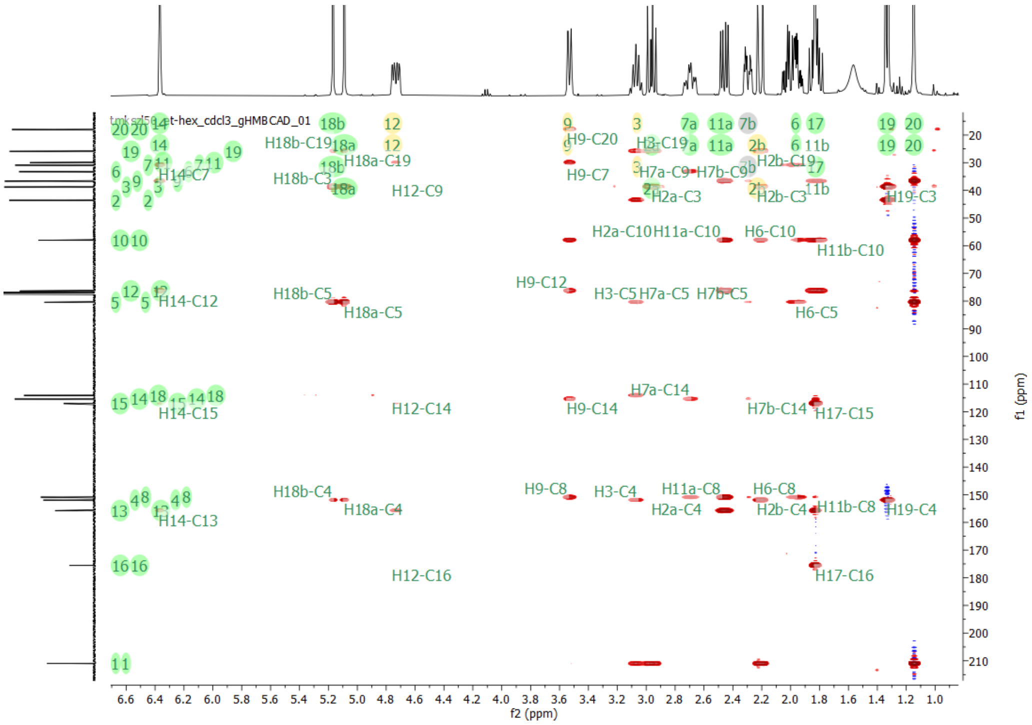

Figure S5: HMBC Spectrum (400/100 MHz, $CDCl_3$, 25 ºC) of Zanzibariolide A (**1**)

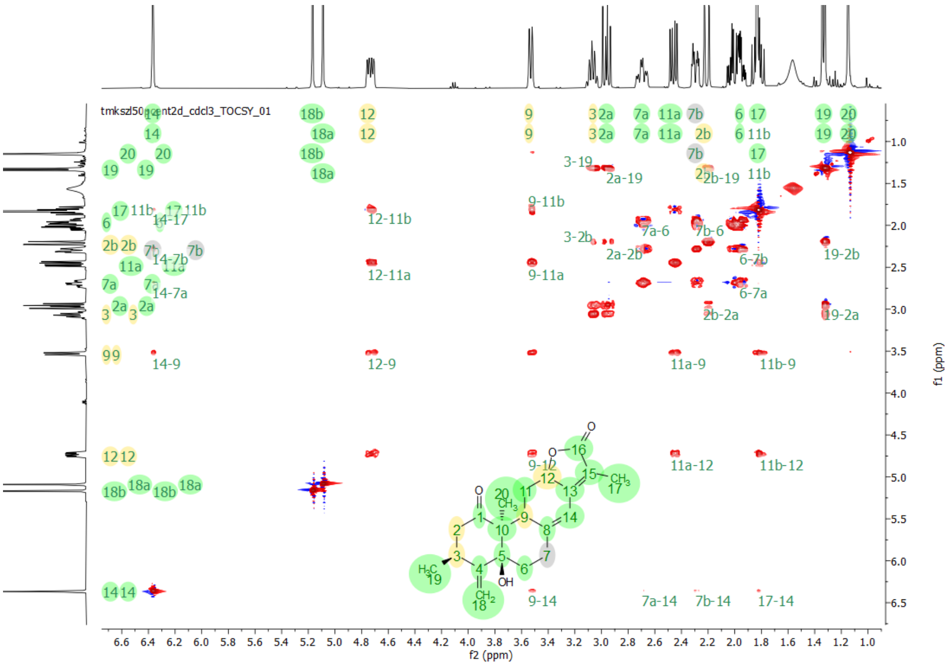

Figure S6: TOCSY Spectrum (400 MHz, $CDCl_3$, 25 ºC) of Zanzibariolide A (**1**)

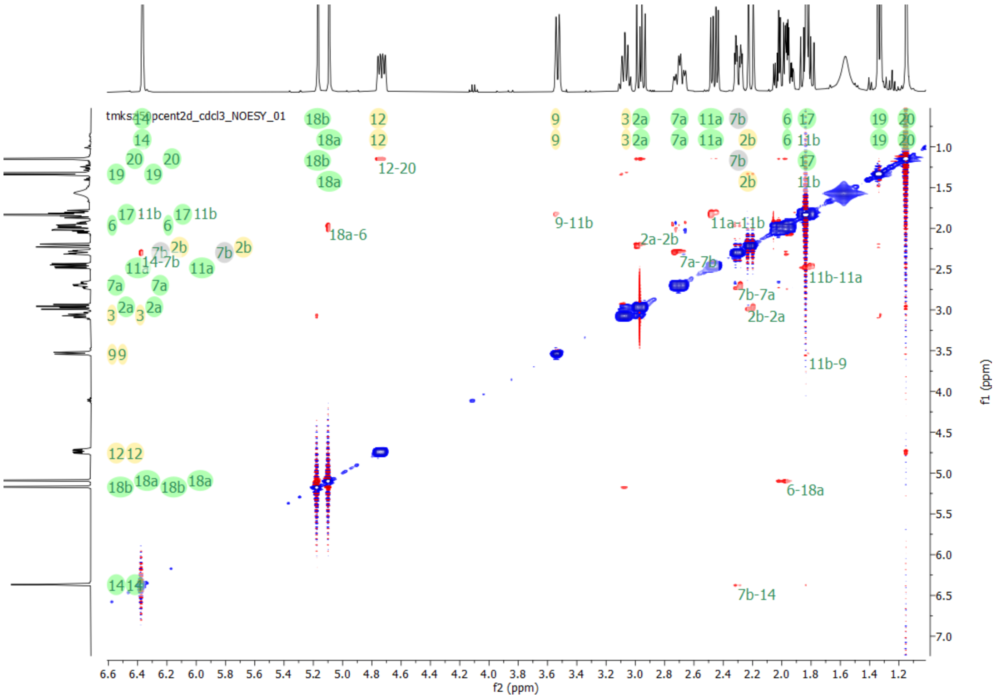

Figure S7: NOESY Spectrum (400 MHz, $CDCl_3$, 25 ºC) of Zanzibariolide A (**1**)

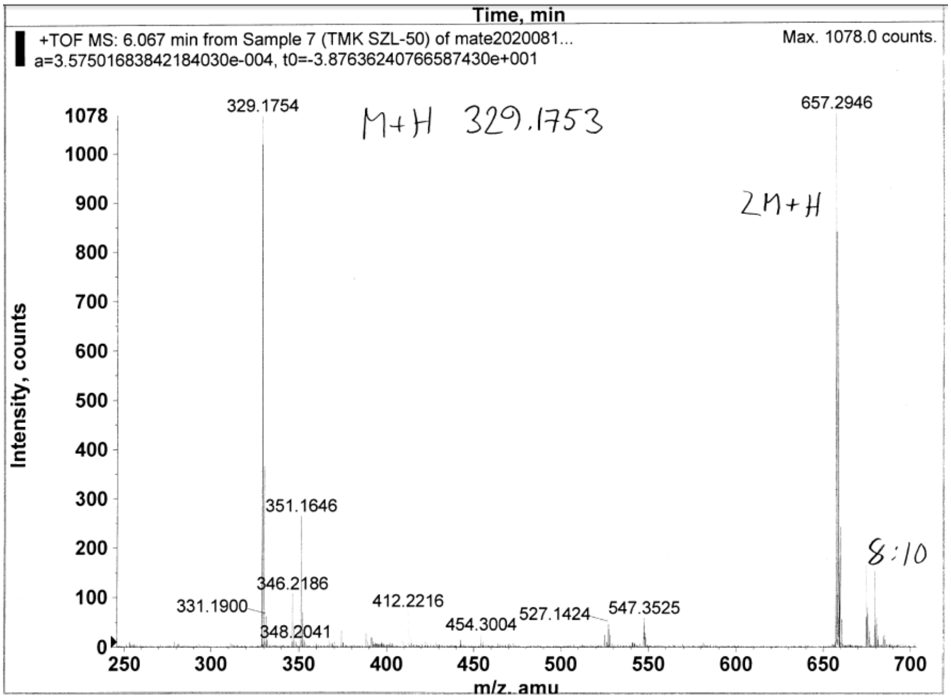

Figure S8: HRESIMS of Zanzibariolide A (**1**)

3. Spectroscopic data of zanzibariolide B (2)

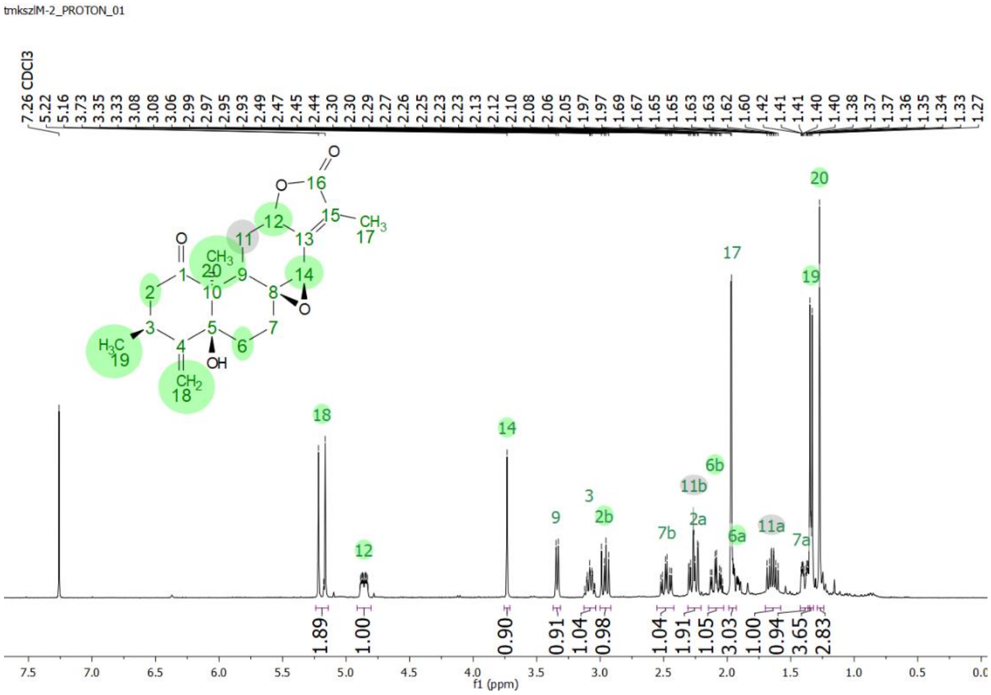

Figure S9: ^{1}H NMR Spectrum (400 MHz, $CDCl_3$, 25 ºC) of Zanzibariolide B (**2**)

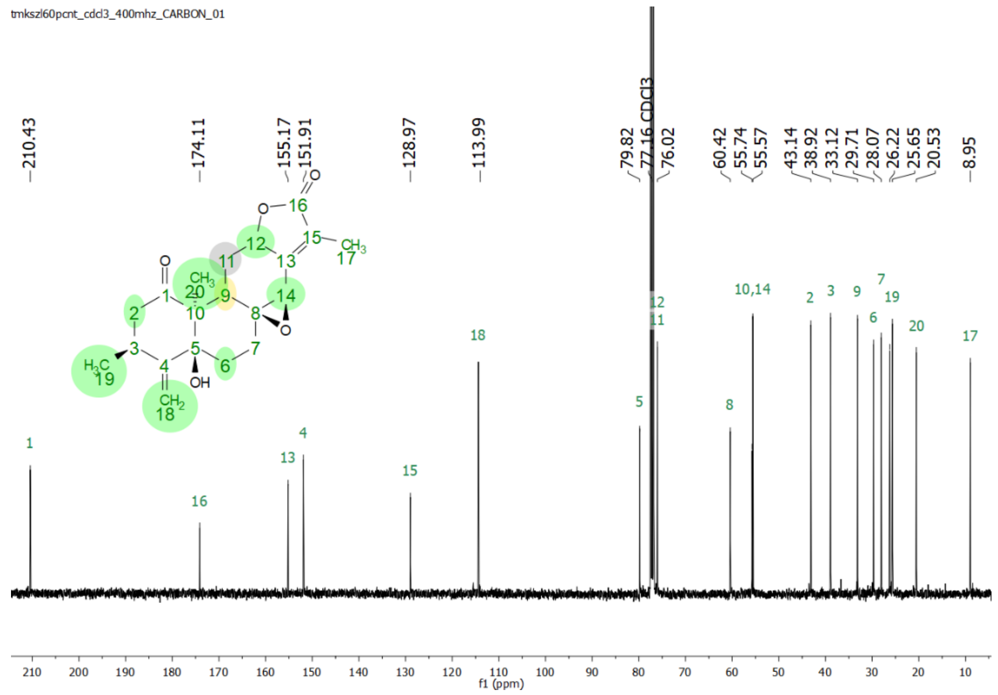

Figure S10: ^{13}C NMR Spectrum (100 MHz, $CDCl_3$, 25 ºC) of Zanzibariolide B (**2**)

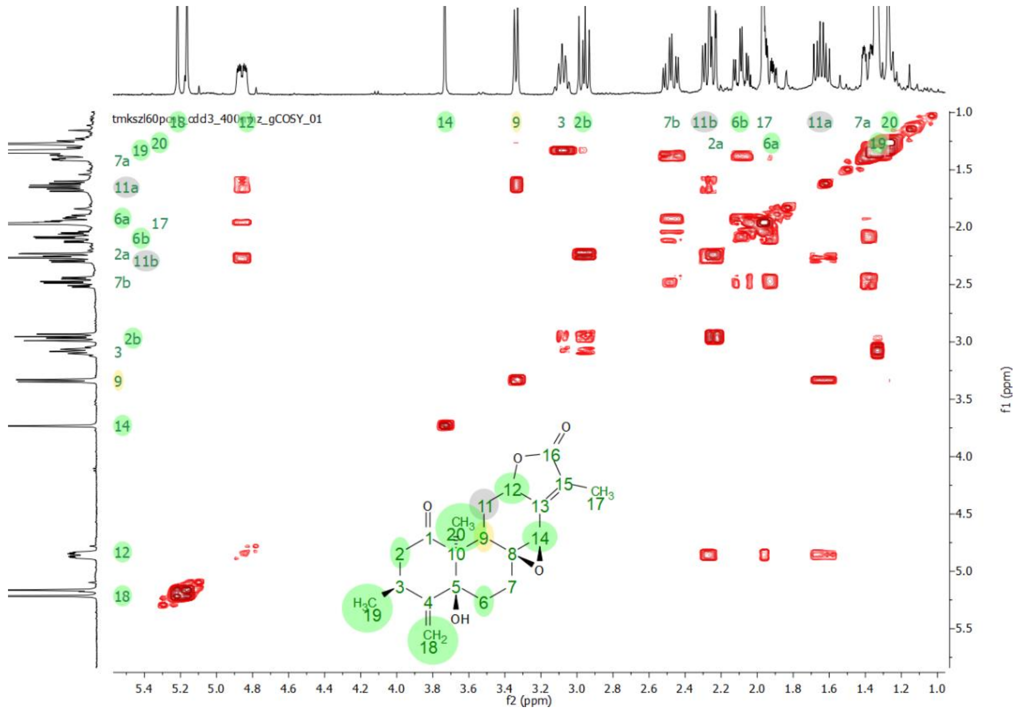

Figure S11: COSY Spectrum (400 MHz, $CDCl_3$, 25 ºC) of Zanzibariolide B (**2**)

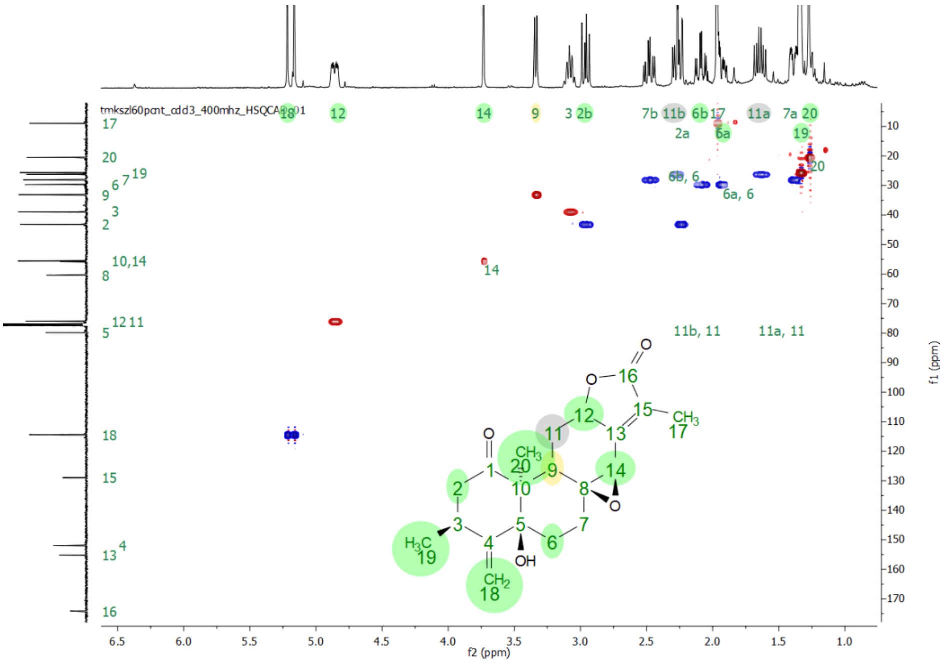

Figure S12: HSQC Spectrum (400/100 MHz, $CDCl_3$, 25 ºC) of Zanzibariolide B (**2**)

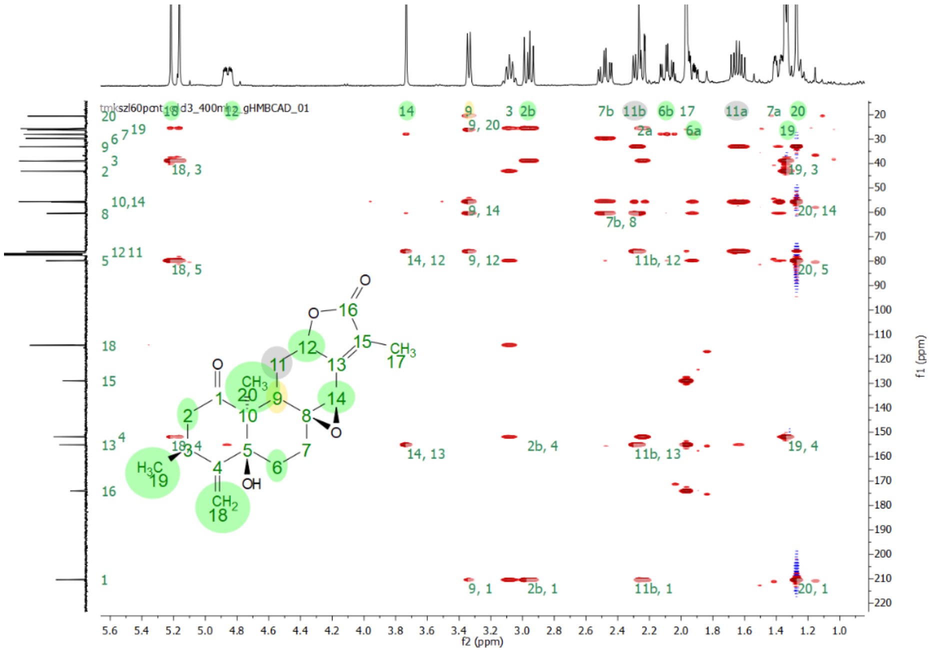

Figure S13: HMBC Spectrum (400/100 MHz, $CDCl_3$, 25 ºC) of Zanzibariolide B (**2**)

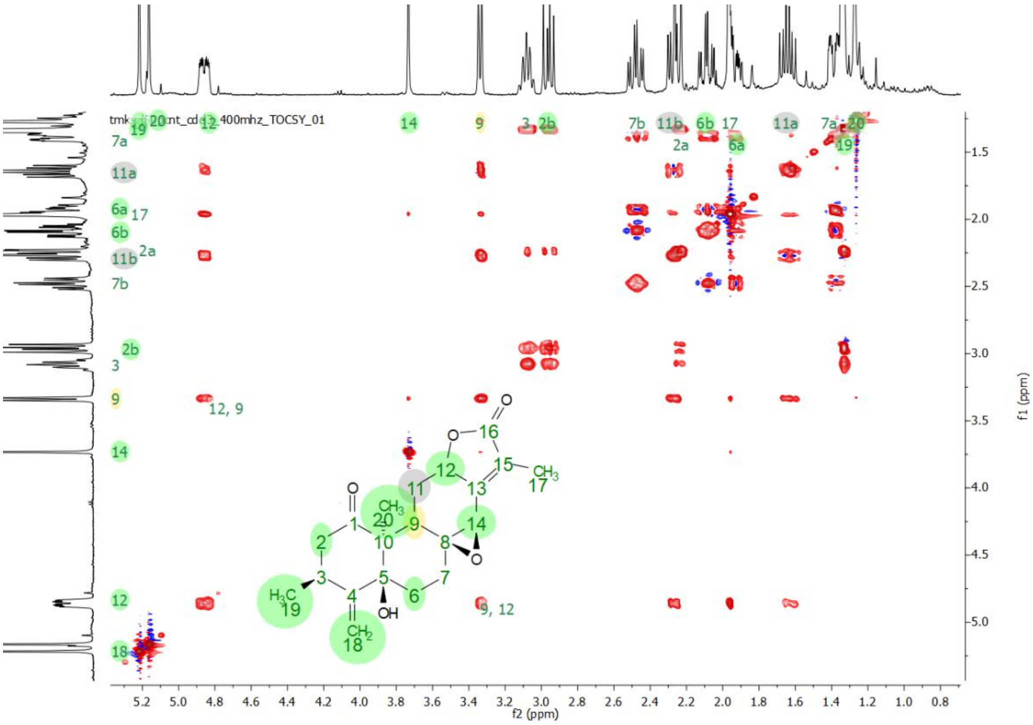

Figure S14: TOCSY Spectrum (400 MHz, $CDCl_3$, 25 ºC) of Zanzibariolide B (**2**)

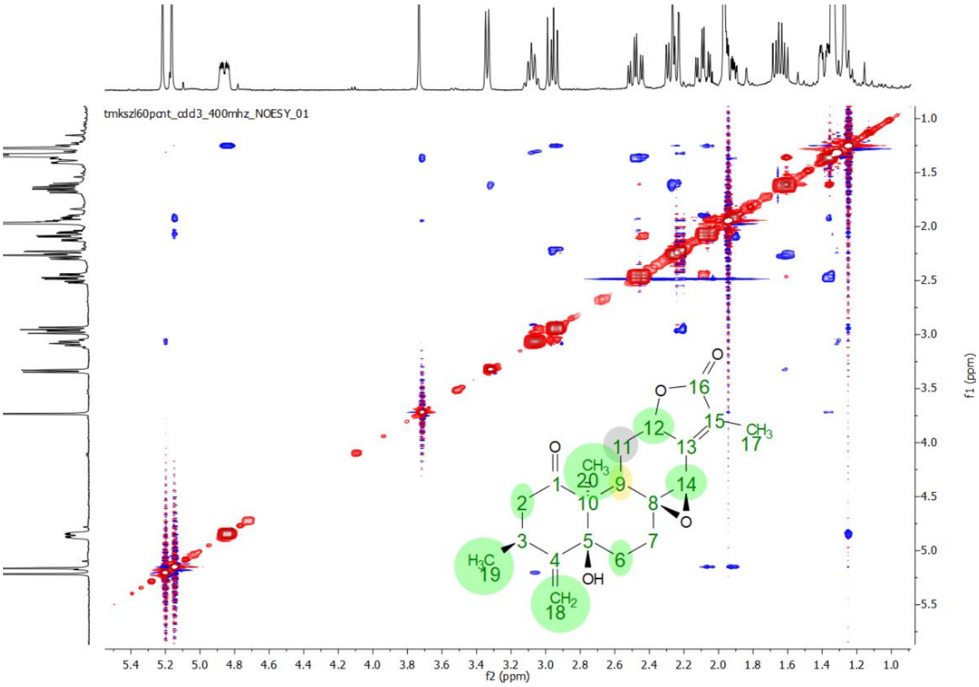

Figure S15: NOESY Spectrum (400 MHz, $CDCl_3$, 25 ºC) of Zanzibariolide B (**2**)

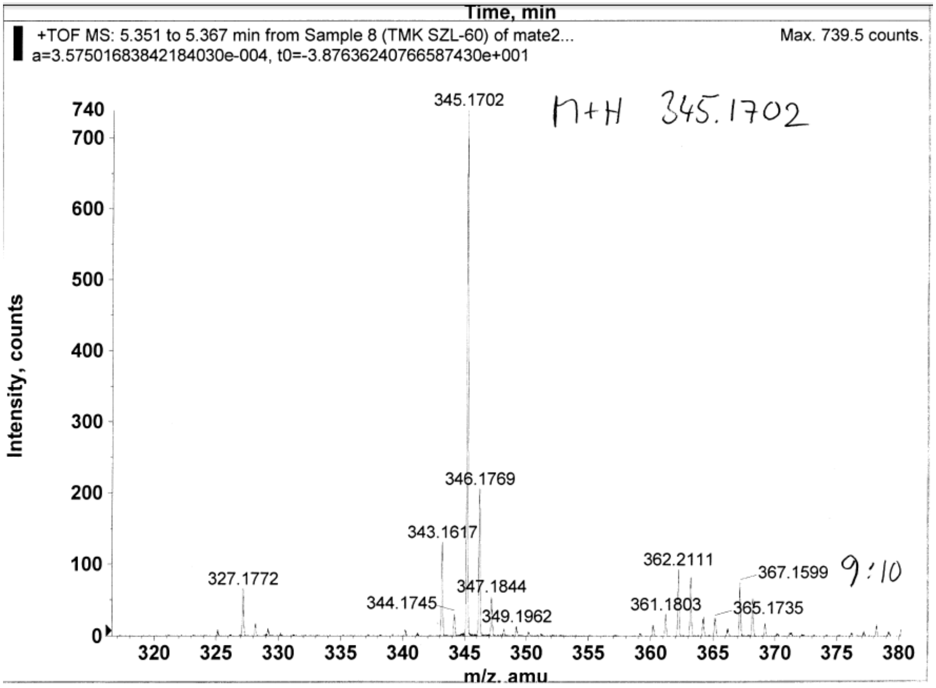

Figure S16: HRESIMS of Zanzibariolide B (**2**)

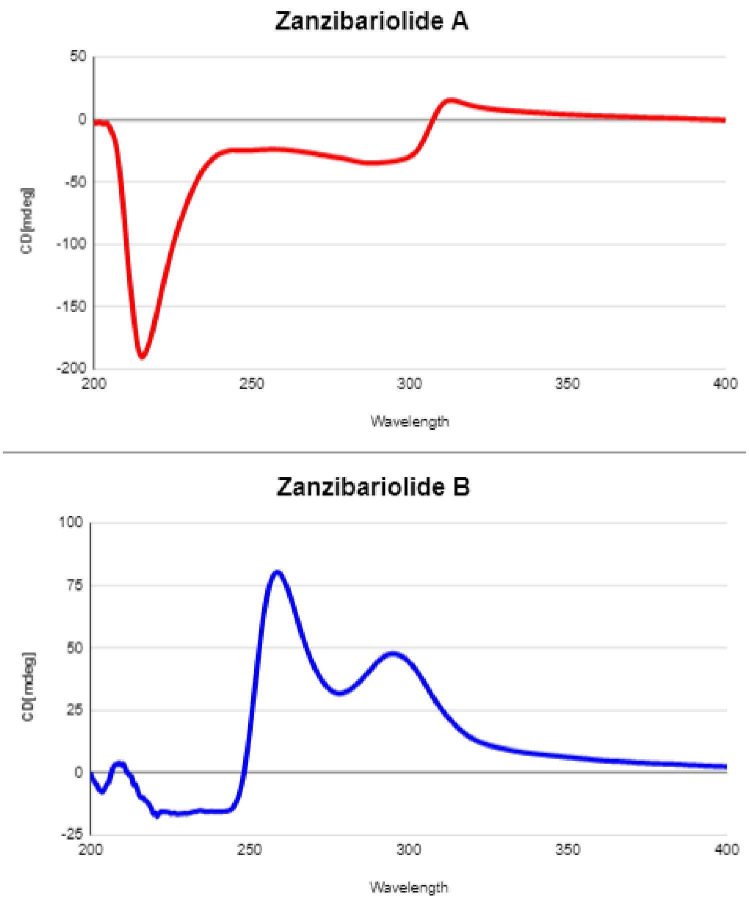

Figure S17: ECD spectra of Zanzibariolide A (**1**, **red**), Zanzibariolide B (**2**, **blue**), in CH_2Cl_2

Table S1. HMBC ($^1H \rightarrow ^{13}C$) of 1 and 2 (400/100 MHz, $CDCl_3$, 25 ºC)

	**1**<br>HMBC, H→C	**2**<br>HMBC, H→C
1		
2	C1, C-3, C-4, C-10, C-19<br>C1, C-3, C-4, C-10, C-19	C-1, C-4, C-10, C-19<br>C-1, C-4, C-10, C-19
3	C-18, C-1, C-5	C-2, C-18, C-19
4		
5		
6	C-4, C-8, C-10<br>C-4, C-8, C-10	C-5, C-7, C-8, C-10<br>C-5, C-7, C-8, C-10
7	C-5, C-8, C-9, C-14<br>C-5, C-8, C-9, C-14	C-5, C-9, C-14<br>C-5, C-9, C-14
8		
9	C-1, C-12, C-7, C-14	C-1, C-5, C-12, C-14, C-20
10		
11	C-8, C-10, C-13<br>C-8, C-10, C-13	C-8, C-13, C-10<br>C-8, C-13, C-10
12	C-9, C-13, C-14, C-15	C-9, C-13, C-14, C-15,
13		
14	C-7, C-9, C-12. C-13, C-15	C-12, C-13
15		
16		
17	C-13, C-16	C-13, C-15, C-16
18	C-3, C-4, C-5<br>C-3, C-4, C-5	C-3, C-4, C-5<br>C-3, C-4, C-5
19	C-2, C-3, C-4	C-2, C-4, C-3
20	C-1, C-5, C-9, C-10	C-1, C-5, C-10

4. Spectroscopic data of Simiarenol (**3**)

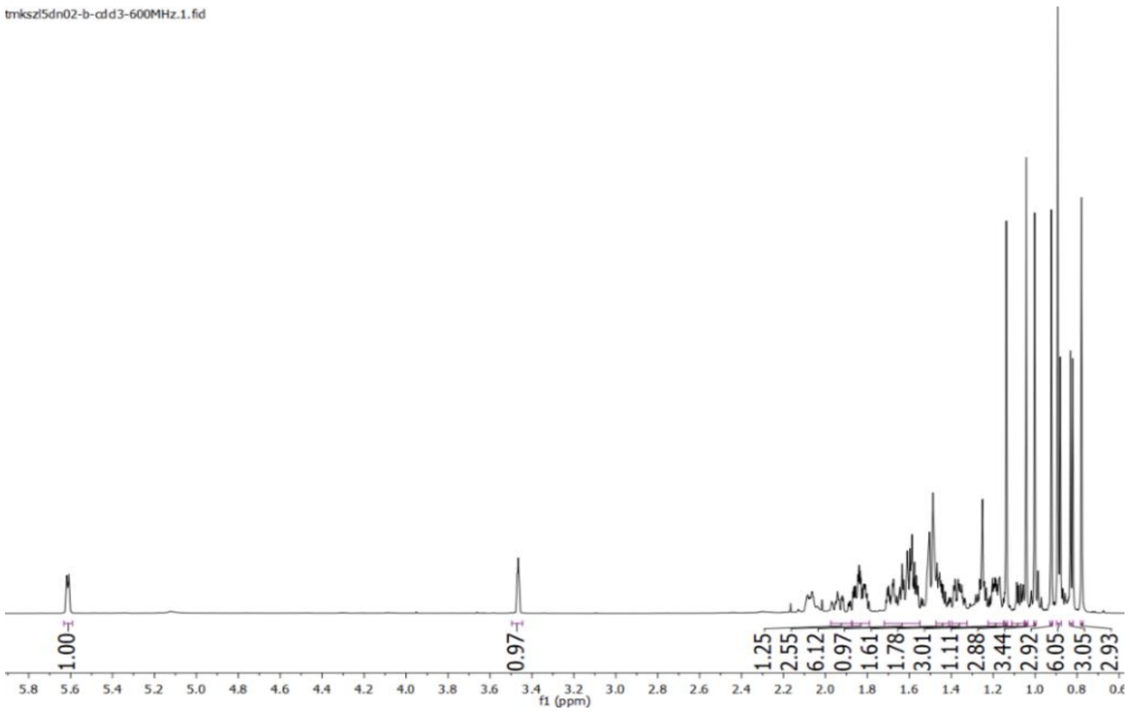

Figure S18: ^{1}H NMR Spectrum (600 MHz, $CDCl_3$, 25 ºC) of Simiarenol (**3**)

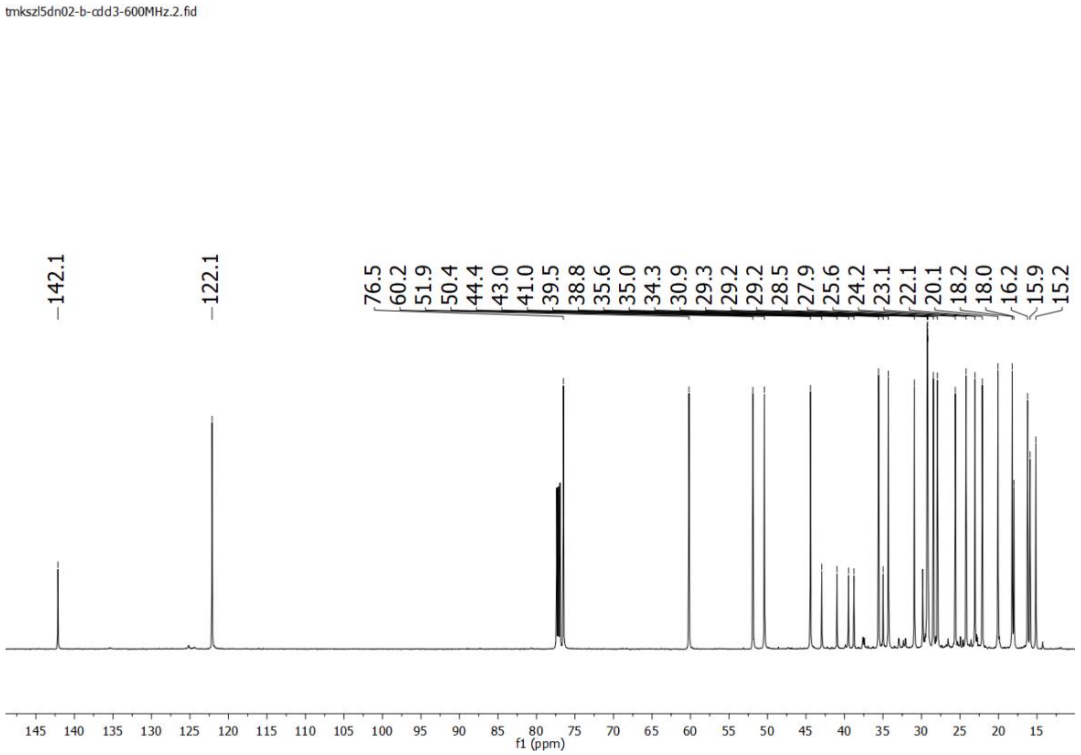

Figure S19: ^{13}C NMR Spectrum (150 MHz, $CDCl_3$, 25 ºC) of Simiarenol (**3**)

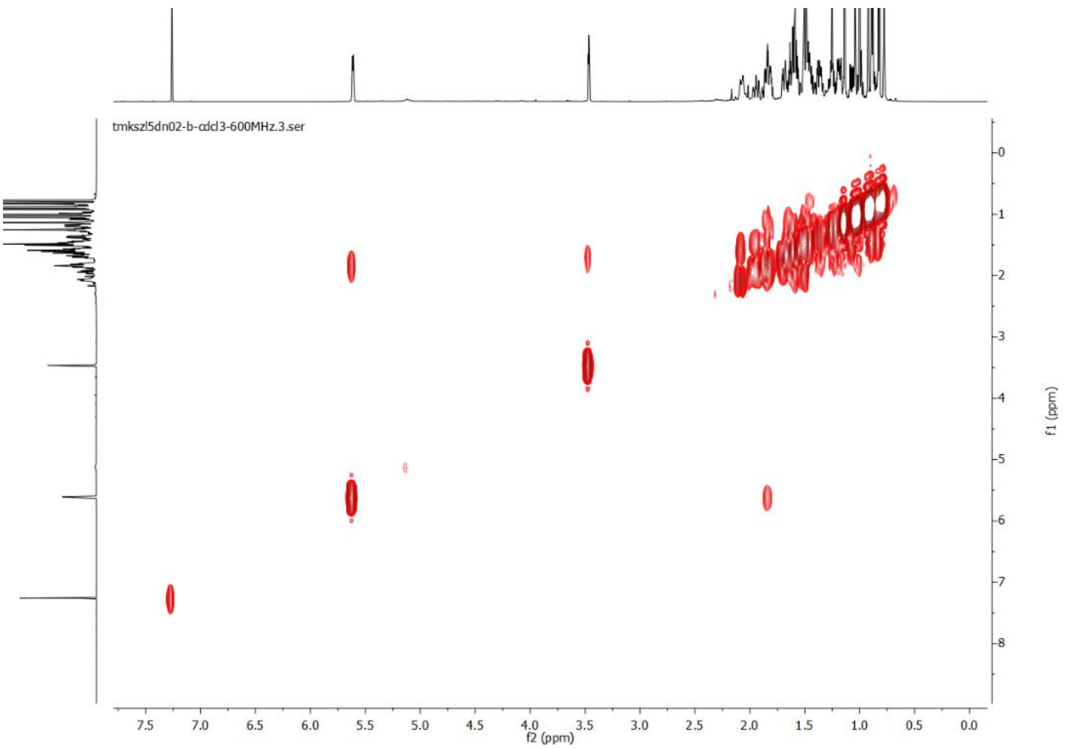

Figure S20: COSY Spectrum (600 MHz, $CDCl_3$, 25 ºC) of Simiarenol (**3**)

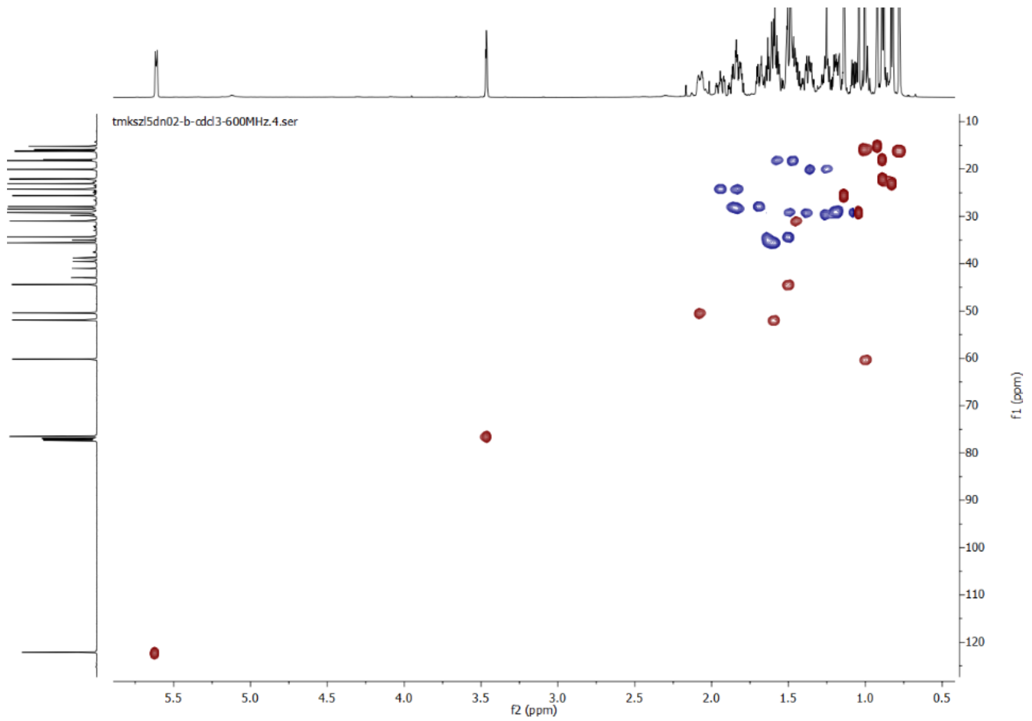

Figure S21: HSQC Spectrum (600/150 MHz, $CDCl_3$, 25 ºC) of Simiarenol (**3**)

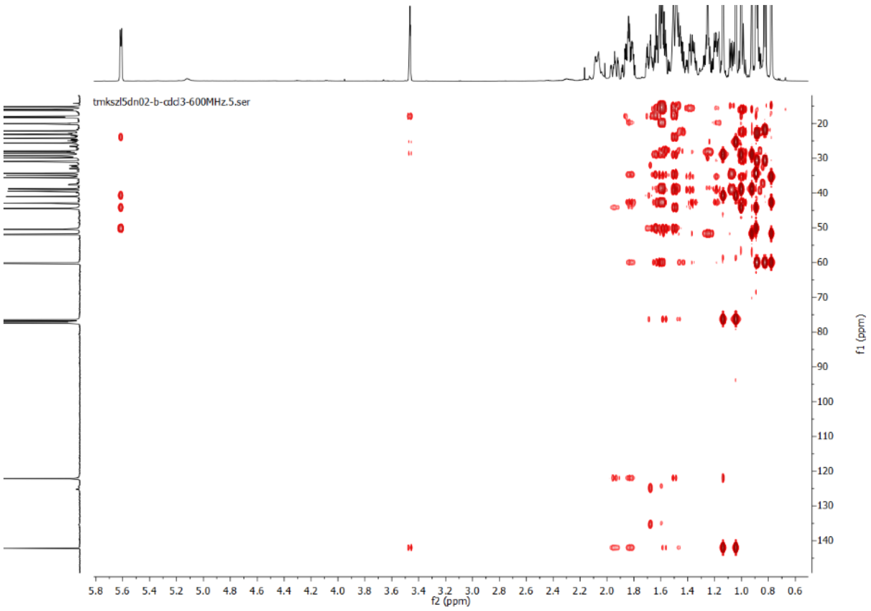

Figure S22: HMBC Spectrum (600/150 MHz, $CDCl_3$, 25 ºC) of Simiarenol (**3**)

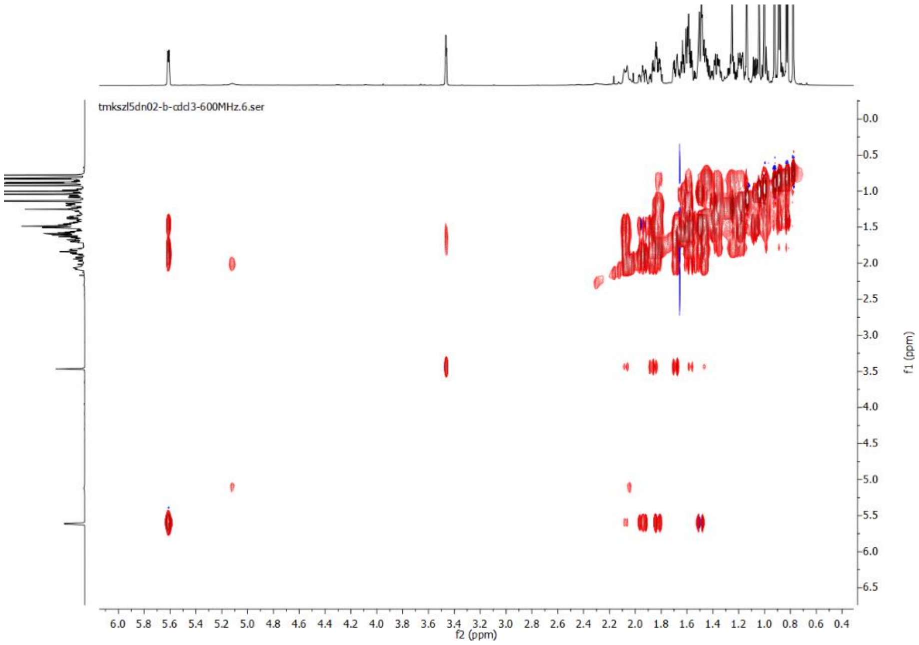

Figure S23: TOCSY Spectrum (600 MHz, $CDCl_3$, 25 ºC) of Simiarenol (**3**)

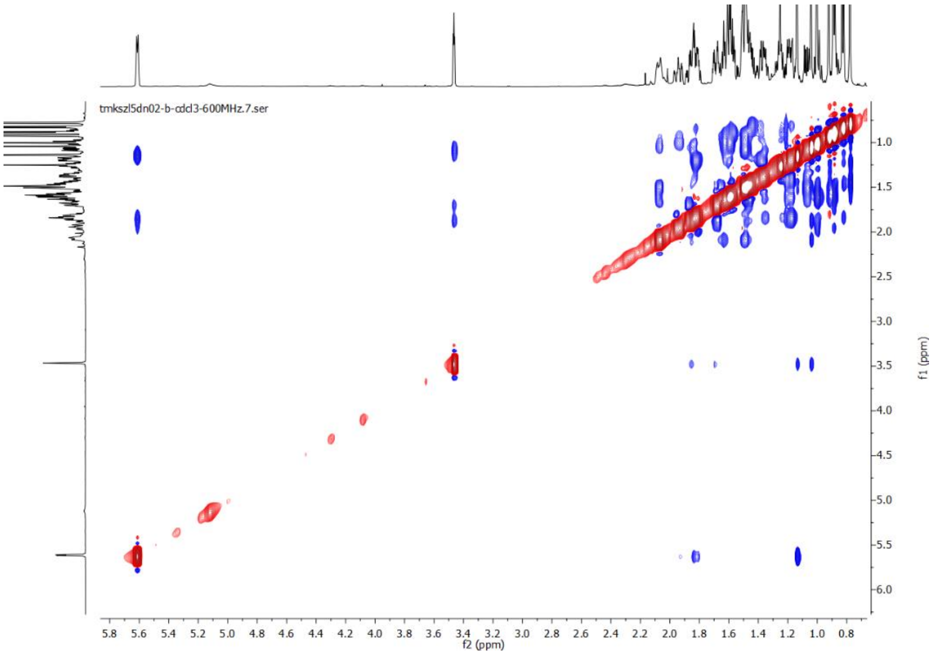

Figure S24: NOESY Spectrum (600 MHz, $CDCl_3$, 25 ºC) of Simiarenol (**3**)

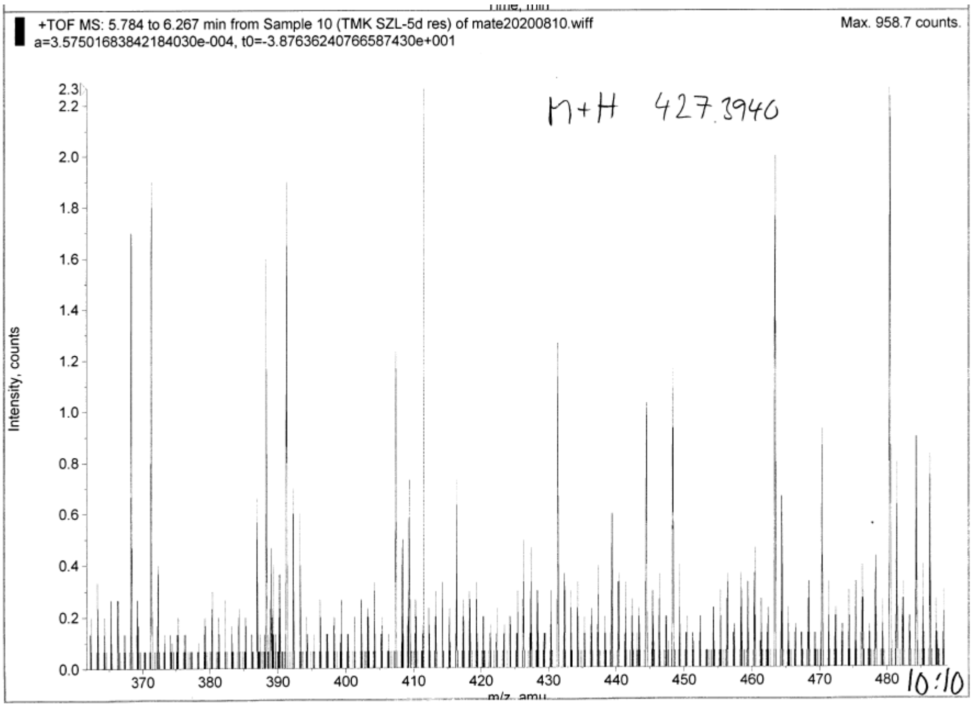

Figure S25: HRESIMS of Simiarenol (**3**)

5. Spectroscopic data of β-amyrin **(4)**

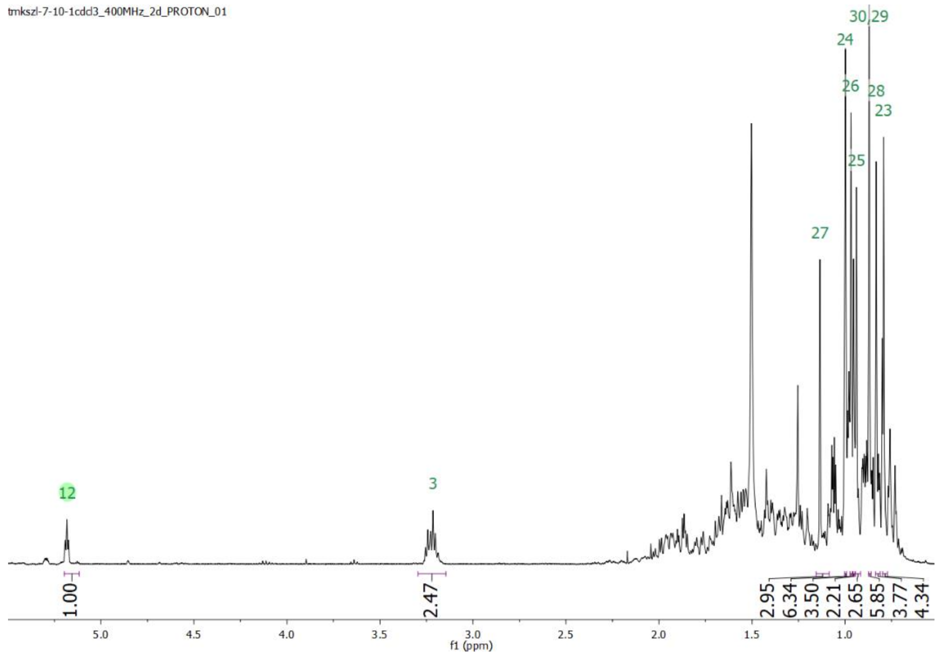

Figure S26: ^{1}H NMR Spectrum (400 MHz, $CDCl_3$, 25 ºC) of *β*-Amyrin (**4**)

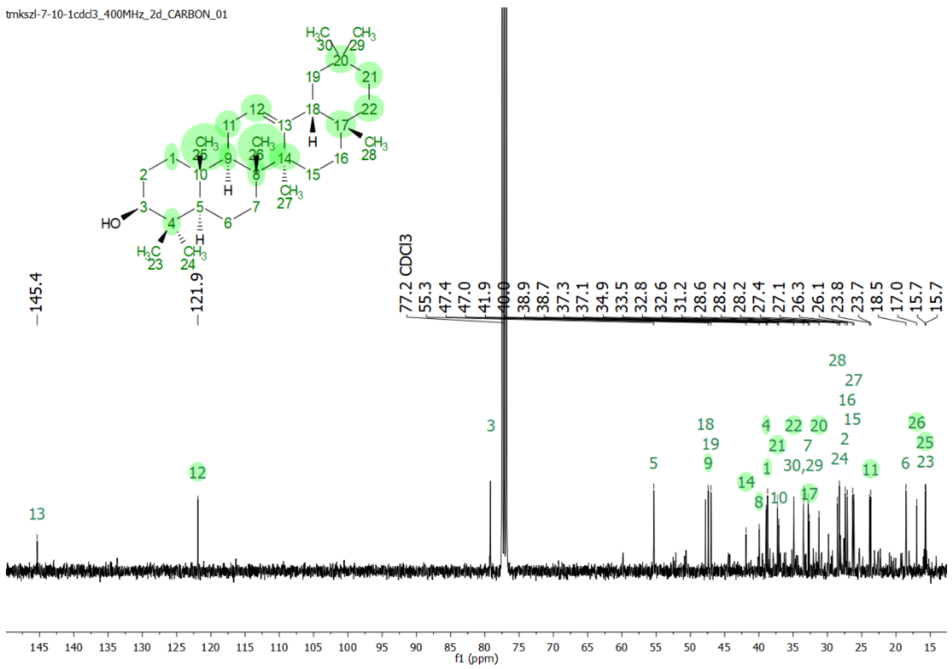

Figure S27: ^{13}C NMR Spectrum (100 MHz, $CDCl_3$, 25 ºC) of *β*-Amyrin (**4**)

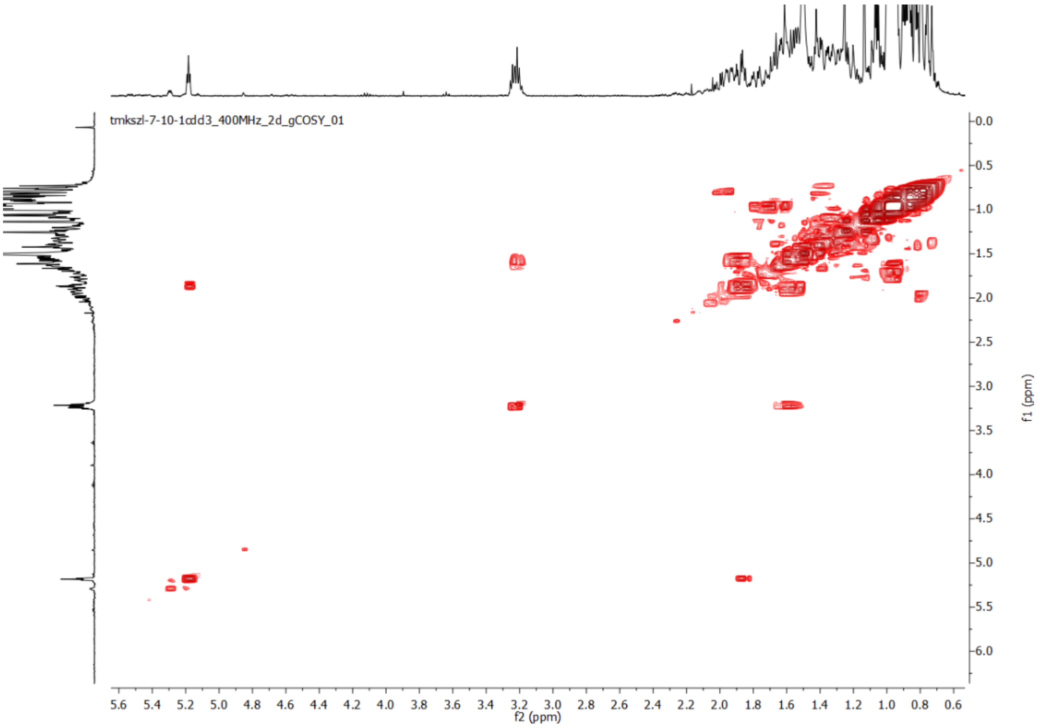

Figure S28: COSY Spectrum (400 MHz, $CDCl_3$, 25 ºC) of *β*-Amyrin (**4**)

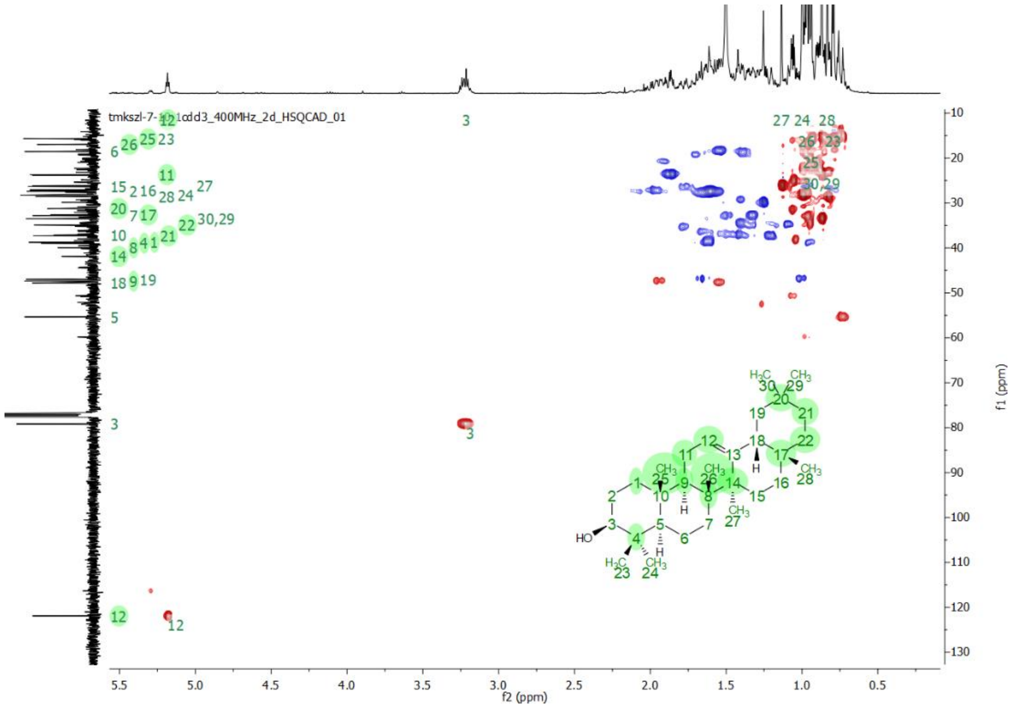

Figure S29: HSQC Spectrum (400/100 MHz, $CDCl_3$, 25 ºC) of *β*-Amyrin (**4**)

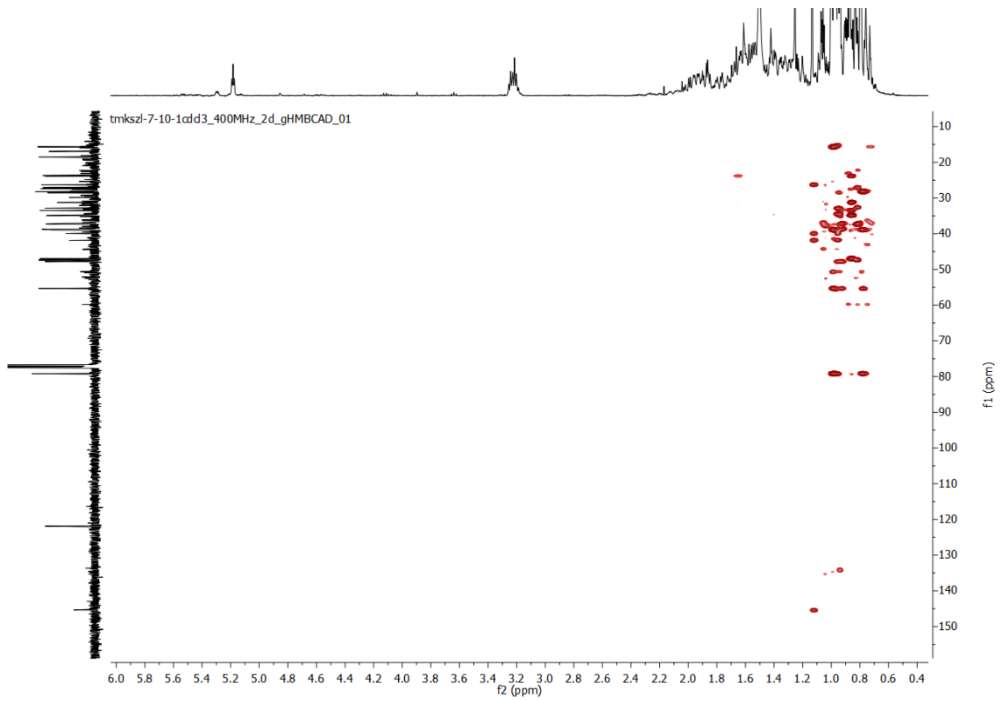

Figure S30: HMBC Spectrum (400/100 MHz, $CDCl_3$, 25 ºC) of *β*-Amyrin (**4**)

Table S2: ^{1}H/ ^{13}C NMR Spectroscopic Data (600 MHz, $CDCl_3$, 25 ºC) for Simiarenol (**3**)

**position**	**δ_C, type**	**δ_H (*J* in Hz)**	**HMBC, H→C**
1	18.2, CH_2	1.46, *m*, 1.57, *m*	C-3, C-5, C-9
2	27.9, CH_2	1.69, *m*, 1.84, *m*	C-4, C-10<br>C-4, C-10
3	76.5, CH	3.47, *dd* (3.7, 2.3)	C-1, C-5
4	41.0, C	-	
5	142.1, C	-	
6	122.1, CH	5.60, *dt* (6.0, 2.2, 2.2)	C-4, C-8, C-10
7	24.2 CH_2	1.83, *m*<br>1.93, *m*	C-5, C-9, C-14<br>C-5, C-9, C-14
8	60.4, C	1.49, *m*	C-6, C-10, C-13
9	35.0, C	-	
10	50.4, CH	2.07, *m*	C-2, C-4,C-6, C-8
11	34.8, CH_2	1.63, *m*<br>1.50, *m*	C-10, C-8, C-13<br>C-10, C-8, C-13
12	29.2, CH_2	1.18, *m*	C-9, C-14, C-18
13	38.8, C	-	
14	39.5, C	-	
15	29.2, CH_2	1.37, *m*<br>1.48, *m*	C-13, C-8, C-17<br>C-13, C-8, C-17
16	35.6, CH_2	1.59, *m*	C-14, C-18, C-21
17	43, C	-	
18	51.9, CH	1.60, *m*	C-14, C-20, 28
19	20.1, CH_2	1.24, *m*, 1.37, *m*	C-13, C-17, C-21
20	28.5, CH_2	1.84, *m*	C-17, C-18, C-22
21	60.2, CH	0.99, *m*	C-19, C-30, C-29
22	30.9, CH	1.45, *m*	C-17, C-20
23	29.1, CH_3	1.04, *s*	C-3, C-5
24	25.6, CH_3	1.14, *s*	C-3, C-5
25	18.0, CH_3	0.78, *s*	C-10, C-11
26	15.9, CH_3	0.89, *s*	C-13
27	15.2, CH_3	0.92, *s*	C-14
28	16.2, CH_3	1.00, *s*	C-18
29	22.1, CH_3	0.83, *s*	C-21, C-30
30	23.1, CH_3	0.88, *s*	C-21, C-29

Table S3: $^{1}H/^{13}C$ NMR Spectroscopic Data (400 MHz, $CDCl_3$, 25 ºC) for *β*-Amyrin (**4**)

**position**	**δ_C, type**	**δ_H (*J* in Hz)**	**HMBC, H→C**
1	38.7, CH_2	1.57, *m*	C-3, C-5, C-9
2	27.4, CH_2	1.60, *m*	C-4, C-10
3	79.2, CH	3.23, *dd* (3.7, 5.1)	C-1, C-5
4	38.9, C	-	
5	55.3, CH	0.73, *m*	C-1, C-3
6	18.5, CH_2	1.56, *m*<br>1.37, *m*	C-4, C-8, C-10<br>C-4, C-8, C-10
7	32.8, CH_2	0.90, *m*	C-5, C-9, C-14
8	40.0, CH	-	
9	47.4, CH	1.96, *m*	C-5, C-12, C-14
10	37.1, C	2.07, *m*	C-2, C-4, C-6, C-8
11	23.7, CH_2	1.87, *m*	C-8, C-10, C-13
12	121.9, CH	5.19, *m*	C-9, C-14, C-18
13	145.4, C	-	
14	41.9, C	-	
15	26.3, CH_2	0.98, *m*<br>0.82, *m*	C-8, C-13, C-17<br>C-13, C-8, C-17
16	27.1, CH_2	1.60, *m*	C-14, C-18, C-28
17	32.7, C	-	
18	47.8, CH	1.55, *s*	C-12, C-14, C-20, C-28,
19	47.0, CH_2	1.66, *m*<br>1.00, *m*	C-13, C-21, C-17, C-29, C-30
20	28.5, C	-	
21	37.1, CH_2	1.42, *m*<br>1.21, *m*	C-17, C-18, C-30, C-29
22	34.9, CH_2	1.41, *m*<br>1.08, *m*	C-18, C-20<br>C-18, C-20
23	15.7, CH_3	0.79, *s*	C-3, C-5
24	24.2, CH_3	1.00, *s*	C-3, C-5
25	15.2, CH_3	0.94, *s*	C-1, C-5, C-9,
26	17.0, CH_3	0.97, *s*	C-7, C-9, C-14
27	26.1, CH_3	1.13, *s*	C-8, C-13
28	28.6, CH_3	0.83, *s*	C-18, C-22
29	33.5, CH_3	0.87, *s*	C-19, C-21, C-30,
30	23.8, CH_3	0.87, *s*	C-19, C-21, C-29,

6. X-ray crystallography of Zanzibariolide A (**1**) and B (**2**), and Simiarenol (**3**)

***Crystal structure determination of Zanzibariolide A (1)*:** $C_{20}H_{24}O_4$, M = 328.39, colourless block, 0.24 x 0.27 x 0.37 mm, orthorhombic, space group *P*$2_12_12_1$, a = 8.4509(1) Å, b = 10.7590(1) Å, c = 18.4968(2) Å, V = 1681.79(3) Å^3, Z = 4, D_{calc} = 1.297 gcm^{-3}, F000 = 704, μ = 0.72 mm^{-1}, T = 120.0(1) K, θ_{max} = 76.4°, 3400 total reflections, 3328 with $I_o > 2\sigma(I_o)$, R_{int} = 0.020, 3400 data, 223 parameters, no restraints, GooF = 1.03, $R_1[I_o > 2\sigma(I_o)]$ = 0.028 and wR_2 = 0.075, 0.22 < dΔρ < -0.14 eÅ^{-3}, Flack = 0.07(6), CCDC-2181946. An ORTEP image of the molecule of zanzibariolide A is shown below.

Datablock 1 - ellipsoid plot

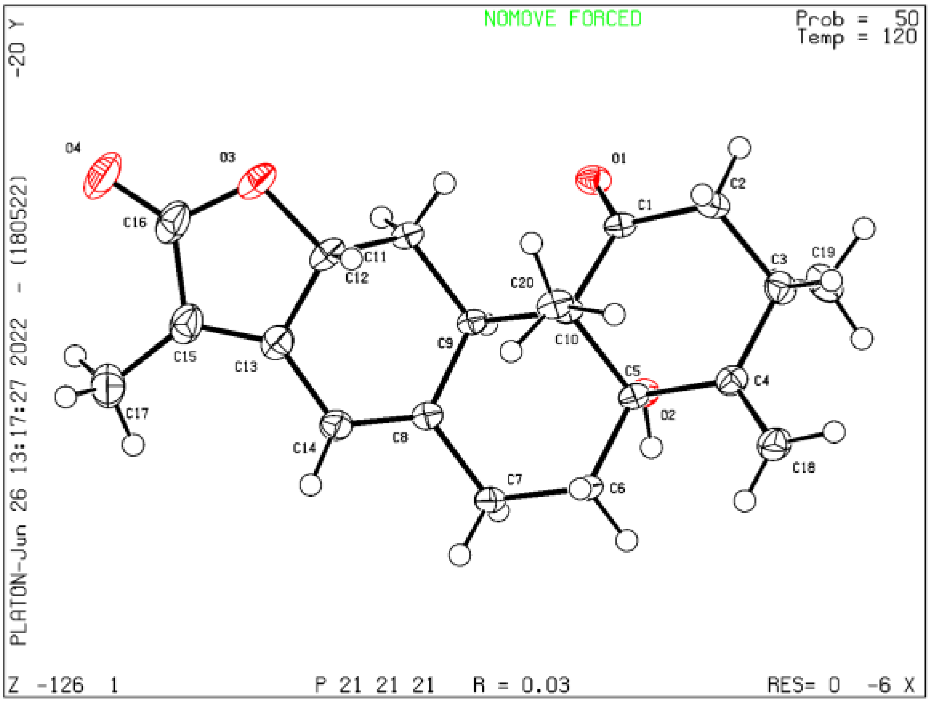

Figure S31 ORTEP drawing of Zanzibariolide A (**1**)

Crystal structure determination of Zanzibariolide B (2): $C_{20}H_{24}O_5$, M = 344.39, colourless plate, 0.06 x 0.18 x 0.24 mm, monoclinic, space group *P*2_1, a = 6.2085(1) Å, b = 16.2459(3) Å, c = 8.2684(1) Å, β = 93.354(2)°, V = 832.54(2) Å^3, Z = 2, D_{calc} = 1.374 gcm^{-3}, F000 = 368, μ = 0.80 mm^{-1}, T = 120.0(1) K, θ_{max} = 76.4°, 3374 total reflections, 3278 with $I_o > 2\sigma(I_o)$, R_{int} = 0.022, 3374 data, 232 parameters, 1 restraint, GooF = 1.05, $R_1[I_o > 2\sigma(I_o)]$ = 0.029 and wR_2 = 0.075, 0.19 < dΔρ < -0.14 eÅ^{-3}, Flack = -0.04(7), CCDC-2181947. An ORTEP image of the molecule of zanzibariolide B is shown below.

Datablock 2 - ellipsoid plot

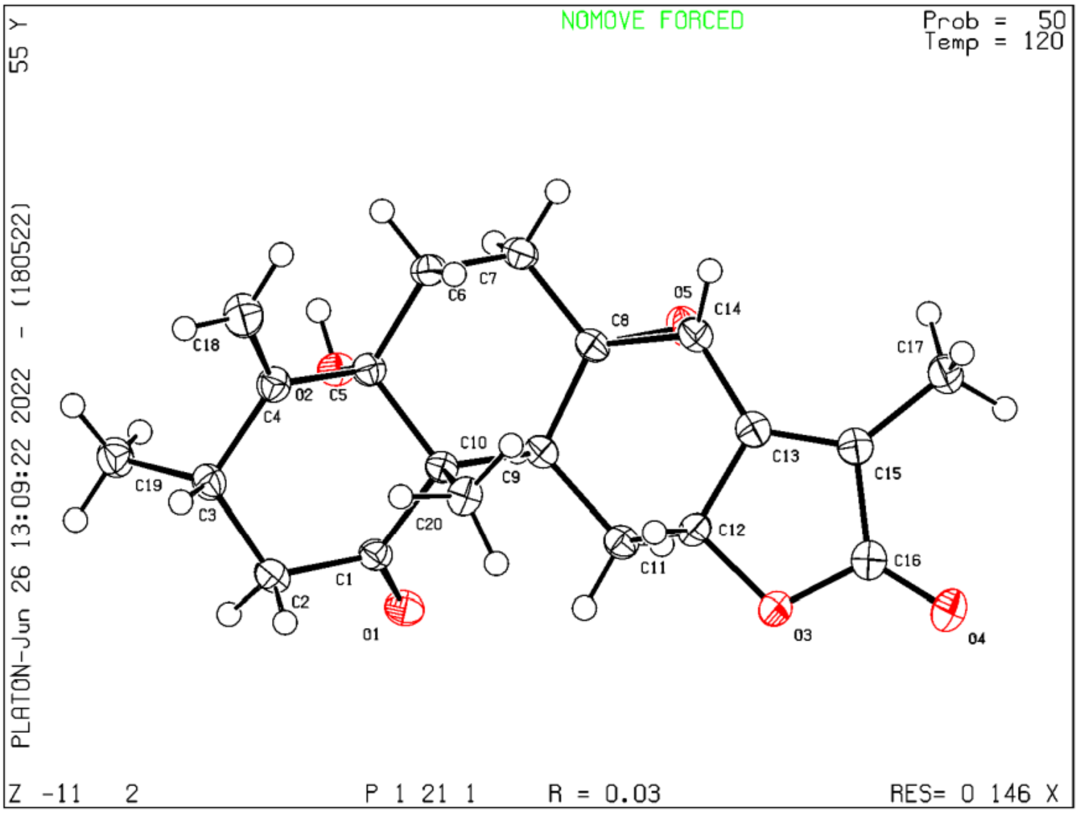

Figure S32 ORTEP drawing of Zanzibariolide B(**2)**

Crystal structure determination of Simiarenol (3): Crystal Data for $C_{30}H_{50}O$ (*M* = 426.70 g/mol): trigonal, space group *R*3 *a* = 35.206(6) Å, *c* = 7.3631(14) Å, *V* = 7903(3) Å^3, *Z* = 9, *T* = 180.15 K, μ(MoKα) = 0.047 mm^{-1}, *Dcalc* = 0.807 g/cm^3, 30808 reflections measured (4.008° ≤ 2Θ ≤ 50.236°), 6228 unique (R_{int} = 0.0808) which were used in all calculations. The final R_1 was 0.0592 (I > 2σ(I)) and wR_2 was 0.1334 (all data). CCDC -2118304.

The X-ray structure data for **1** (CCDC -2181946), **2** (CCDC 2181947) and **3** CCDC 2118304) are deposited with the Cambridge Crystallographic Data Centre. Copies of the data can be obtained, free of charge, on application to Director, CCDC, 12 Union Road, Cambridge CB2 IEZ, UK (fax: +44-(0)1223-336033 or email: deposit@ccdc.cam.ac.uk

7. Anti-tick-borne encephalitis virus (TBEV) and anti-human rhinovirus 2 activity assays

*Determination of anti-tick-borne encephalitis virus (TBEV) activity of compounds **1-3***. The test compounds were assayed for their ability to protect human lung adenocarcinoma cells (A549; ATCC CCL-185) against TBEV-induced cytopathic effect including induction of vacuolized cells. Briefly, the cells, seeded in 96 well cluster plates the day prior to experiment, were rinsed with 100 µL of Eagle's minimum essential medium (EMEM) supplemented with 2% heat-inactivated fetal calf serum, 1% pest stock and 1% L-glutamine stock (EMEM-M), and 30 µL of fresh EMEM-M medium was added. Then, the cells received 20 µL of serial five-fold dilutions of the test compounds (final concentration ranged from 0.032 to 100 µM), and following 2 h period of incubation at 37°C in humidified atmosphere comprising 5% CO_2 (the CO_2 incubator), 50 µl of EMEM-M medium containing 250 tissue culture infectious doses ($TCID_{50}$) of TBEV strain F7203 was added. After incubation of cells in the CO_2 incubator for 3 days, the cells were fixed with 8% solution of paraformaldehyde and stained with 1% solution of crystal violet.

Results: Inspecting of cells under a microscope revealed that at the concentration range tested (0.032 – 100 µM) compounds **1-3** exhibited no protection of A549 cells against TBEV.

*Determination of anti-human rhinovirus 2 (HRV-2) activity of compounds **1-3***. The test compounds were tested for their ability to protect human cervical cancer cells (HeLa) against human rhinovirus type 2 (HRV-2; ATCC VR-482) induced cytopathic effect. Briefly, the cells, seeded in 96 well cluster plates the day prior to experiment, were rinsed with 100 µL of Eagle's minimum essential medium (EMEM) supplemented with 2% heat-inactivated fetal calf serum, 1% pest stock, 1% L-glutamine stock, 30 mM $MgCl_2$ and 20 mM HEPES, pH7.1 (IM-EMEM-M), and 60 µL of fresh IM-EMEM-M medium was added. Then, the cells received 20 µL of serial five-fold dilutions of the test compounds (final concentration ranged from 0.032 to 100 µM), and following 2 h period of incubation at 34°C in the CO_2 incubator, 20 µl of IM-EMEM-M medium containing 100 $TCID_{50}$ of HRV-2 was added. After incubation of cells in the CO_2 incubator for 3 days, the cells were stained with 1% solution of crystal violet.

Results: Inspecting of cells under a microscope revealed that at the concentration range tested (0.032 – 100 µM) compounds **1-3** exhibited no protection of HeLa cells against HRV-2.
